# Modeling of genetic gain for single traits from marker-assisted seedling selection in clonally propagated crops

**DOI:** 10.1038/hortres.2016.15

**Published:** 2016-04-20

**Authors:** Sushan Ru, Craig Hardner, Patrick A Carter, Kate Evans, Dorrie Main, Cameron Peace

**Affiliations:** 1 Department of Horticulture, Washington State University, PO Box 646414, Pullman, WA 99164-6414, USA; 2 Queensland Alliance for Agriculture and Food Innovation, University of Queensland, St Lucia, Brisbane 4072, Australia; 3 School of Biological Sciences, Washington State University, Pullman, WA 99164-4236, USA; 4 Department of Horticulture, Washington State University Tree Fruit Research and Extension Center, Wenatchee, WA 98801, USA

## Abstract

Seedling selection identifies superior seedlings as candidate cultivars based on predicted genetic potential for traits of interest. Traditionally, genetic potential is determined by phenotypic evaluation. With the availability of DNA tests for some agronomically important traits, breeders have the opportunity to include DNA information in their seedling selection operations—known as marker-assisted seedling selection. A major challenge in deploying marker-assisted seedling selection in clonally propagated crops is a lack of knowledge in genetic gain achievable from alternative strategies. Existing models based on additive effects considering seed-propagated crops are not directly relevant for seedling selection of clonally propagated crops, as clonal propagation captures all genetic effects, not just additive. This study modeled genetic gain from traditional and various marker-based seedling selection strategies on a single trait basis through analytical derivation and stochastic simulation, based on a generalized seedling selection scheme of clonally propagated crops. Various trait-test scenarios with a range of broad-sense heritability and proportion of genotypic variance explained by DNA markers were simulated for two populations with different segregation patterns. Both derived and simulated results indicated that marker-based strategies tended to achieve higher genetic gain than phenotypic seedling selection for a trait where the proportion of genotypic variance explained by marker information was greater than the broad-sense heritability. Results from this study provides guidance in optimizing genetic gain from seedling selection for single traits where DNA tests providing marker information are available.

## Introduction

**Figure 1:**
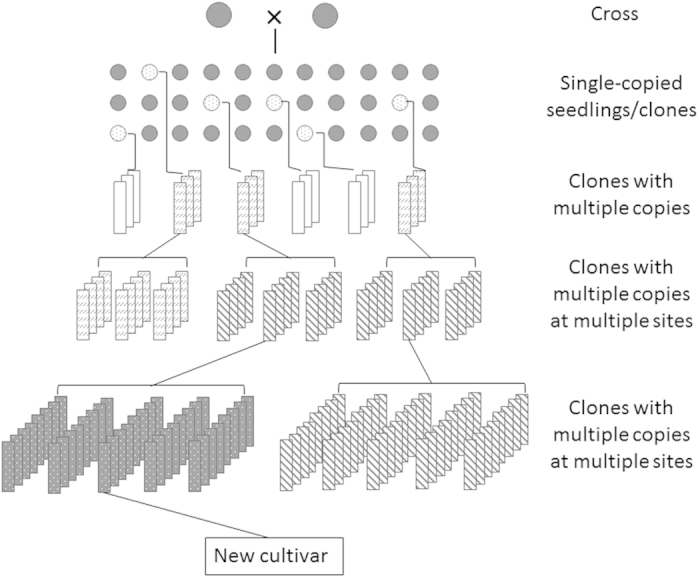
A generalized breeding scheme for clonally propagated crops (modified from Grüneberg *et al.*^1^).

Clonal propagation is routinely used for commercial deployment of elite germplasm in many economically important crops, such as root and tuber crops (for example, potato, garlic, sweet potato, yam), fruit crops (for example, apple, banana, citrus, grape, strawberry), ornamentals (for example, chrysanthemum, roses, tulip) and many forest trees.^1,2^ As an essential way to genetically improve these crops to meet the demand of both consumers and producers, breeding is becoming even more important under a changing environment and a more competitive global market.^3,4^ Compared with seed propagated crops in which whole plant propagation for replicated breeding trials and commercial deployment relies mainly on sexual reproduction via meiosis, breeding of clonally propagated crops combines both sexual and asexual reproduction (Figure 1). Genetic variation in seedling generations is typically provided via sexual reproduction by crossing parents with complementary features. Successive rounds of performance evaluation and selection are then used to identify offspring with increasingly promising genetic potential for consideration as candidate cultivars (Figure 1). Selected individuals are clonally propagated for subsequent replicated trials and, if publicly released, are clonally propagated on a larger scale for commercial production. In this way, dominance and epistatic genetic action, in addition to additive effects, can be captured in selected individuals for contribution to superior commercial performance.^5,6^

Selection decisions in clonally propagated crops are based on individual or family mean performance, depending on the crop and breeding program.^7^ The first round of selection after making crosses, for identifying candidate cultivars, is typically conducted on single copies, or sometimes multiple copies, of each offspring.^3^ Such offspring can be true seed plants (seedlings) or clones of the original seedling. For simplicity, this phase is referred to in the present study as the ‘seedling selection’ phase, and individual plants (or clonally replicated plants in some programs) in this phase as ‘seedlings’. Seedling selection typically reduces family sizes dramatically—for example, the average cull proportion during seedling selection cumulatively for all traits under consideration in the Washington State University apple breeding program is around 98%.^8^ After seedling selection, several additional rounds of selection are conducted on multiple-copy clones grown and evaluated at one or more sites (Figure 1), to confirm genetically superior individuals for previously evaluated traits and/or to impose selection on further traits.

Performance data used for seedling selection decisions can be obtained in several ways. Traditionally, individual seedlings are evaluated based on their phenotype, which here is termed *phenotype-only* seedling selection (‘traditional seedling selection’ in Ru *et al.*^6^). For clonally propagated crops with long generation cycles (for example, apple, peach, pine and many other tree crops), *phenotype-only* is costly and time-consuming when performance evaluation involves large plants and/or must wait until reproductive maturity.^6,9,10^ Where DNA tests are available for valuable trait levels, breeders have the opportunity to predict the genetic potential of young seedlings based on their genotype at DNA markers associated with trait loci^11,12^ and thus reduce financial and other resource costs of selection by maintaining fewer seedlings for field evaluation.^10^ Marker-assisted seedling selection (MASS) utilizes DNA test results, along with phenotypic information, to select seedlings predicted to be genetically superior.^6,13,14^ Here, *marker-only* seedling selection is defined as where only marker genotypes of young seedlings are used in selection decisions for a trait. *Two-stage* seedling selection is defined as selected plants evaluated phenotypically for a subsequent selection step (typically when seedlings are older and field-planted) (adapted from Lande and Thompson^15^). *Index* seedling selection is defined as both phenotypic and genotypic information about a trait being used simultaneously by weighting phenotypic and marker data according to the estimated contributions to target genetic potential.^15^

Overall efficiency of alternative seedling selection strategies may vary widely among breeding populations, trait genetic architecture, and cost structures of phenotyping and DNA testing activities.^6^ To optimize efficiency in seedling selection, alternative strategies to identify seedlings with superior genetic potential need to be considered based on estimated genetic gain, cost and time,^6^ where genetic gain is defined as the increase in the mean genotypic value of selected individuals compared with all individuals before selection (following Holland *et al.*^5^). The time duration of MASS is the same as *phenotype-only* seedling selection unless field evaluation is substantially reduced or entirely skipped as would occur if most or all traits usually field-evaluated were instead selected in *marker-only*. Cost evaluation of marker-based strategies has been reported in clonally propagated crops—for example, apple, grape and strawberry.^10,16,17^ Such studies identified that cost savings from MASS compared with traditional seedling selection were most likely to be made where selection was conducted on young seedlings of perennial crops.

Previous studies have empirically evaluated genetic gain from marker-assisted (seedling) selection for seed-propagated crops or recurrent selection for clonally propagated crops.^18–22^ Genetic gain from marker-assisted selection was also modeled using analytical derivation^15,23,24^ and stochastic simulation^25–27^ based on additive models. Studies based on additive models suggested that two parameters are important in determining the relative efficiency of marker-based selection strategies compared with phenotypic selection: the proportion of the total additive genetic variance caused by the known loci (*p*) and the narrow-sense heritability of the trait (*h*^*2*^).^15,24,25^ However, conclusions based on additive models were not directly relevant for MASS for clonally propagated crops, because of the total genotypic effects instead of only additive effects captured by clonal propagation. A major challenge of estimating genetic gain for clonally propagated crops is a lack of models suitable for idiosyncrasies of this category of crop.^6^

To optimize genetic gain from seedling selection for clonally propagated crops, models for predicting genetic gain from alternative seedling selection strategies are needed. The objective of this study was to model potential genetic gain from alternative seedling selection strategies to provide guidance in optimizing genetic gain for single target traits in a generalized selection scheme for clonally propagated crops.

## Materials and methods

### Selection strategies

A generalized seedling selection scheme for clonally propagated crops was used in genetic gain modeling (Figure 1). In this scheme, F_1_ seeds were considered to be harvested from one or more bi-parental cross(es) and seedlings were planted without replication. Seedlings were hypothetically selected by one of four alternative strategies: *phenotype-only*, *marker-only*, *two-stage and index*. Set proportions of retained seedlings after selection, referred to as total selection proportion (TSP) were used, to enable fair comparisons among strategies. For each strategy, genetic gains were modeled for TSP values ranging from 0.05 to 0.95, with intervals of 0.05. In *phenotype-only*, seedlings were sorted according to their phenotypic values, and a proportion of seedlings with highest phenotypic values were selected. In *marker-only*, seedlings with highest marker effects were selected. If selection was needed among individuals with the same genotype within a certain TSP, retained individuals were randomly selected. In two-stage, seedlings were first selected based on individuals’ marker genotype, and then remaining seedlings were phenotypically selected. The proportion of seedlings selected in the first stage was referred to as SP_M_, and the proportion of remaining seedlings selected in the second stage was SP_P_ (SP=TSP/SP_M_). For a given TSP, all possible SP_M_ values ranging from TSP to 1 with intervals of 0.05 were modeled. Average genetic gains achieved at each SP_M_ value were compared and optimal genetic gains from two-stage seedling selection were used for comparisons with other strategies. *Index* was simulated by generating a weighted selection index for each individual as:

(1)I=bzz+bmm

where *b*_*z*_ was the weight coefficient of the phenotypic score, and *b*_*m*_ was the weight coefficient of the marker score. The ratio between *b*_*m*_ and *b*_*z*_ was calculated as: *b_m_/b_z_*=(1/*H*−1)/(1−*p*), where *H* was the broad-sense heritability, which equaled the proportion of phenotypic variance (*V*_P_) explained by genotypic variance (*V*_G_): *H*=*V*_G_/*V*_P_; *P* was the predictiveness of the DNA test, which was calculated as the proportion of genotypic variance explained by marker loci used in the DNA test: *P*=*V*_M_/*V*_G_, where *V*_M_ was the variance explained by markers.

### Genetic model

All parameters used in simulation were assumed to be estimated without error and markers used in each DNA test were completely linked to the trait locus or loci. The phenotypic value of an individual (*z*) was defined as:

(2)z=g+e

where *g* was the genotypic value of the individual and *e* was the environmental effect, which was normally distributed with a mean of zero and variance *V*_*E*_. *g* could be further decomposed as:

(3)g=gM+gB

where *g*_M_ was the effect of trait loci explained by the markers in the DNA test, which had a variance *V*_M_. *g*_B_ was the background genotypic effect caused by genotypes at all influencing loci in the genome other than the DNA test-targeted loci, which followed a normal distribution with zero mean and variance *V*_B_. Interactions between trait loci and the background genome were assumed to be 0.

Finally, following Equation (2) the total phenotypic variance could be partitioned as:

(4)VP=VG+VE

Genotypic variance could be partitioned as:

(5)VG=VM+VB

### Segregating populations and trait-test scenarios

Two populations with various segregation patterns for the trait under selection were simulated. The total phenotypic variance, *H*, and predictiveness values, *P*, of the population were assumed to be known exactly. Various trait-test scenarios with unique combinations of *H* and *P* values were simulated to study the influence of *H* and *P* on genetic gain. Each population was assigned the distribution of marker genotypes. Seedlings from each population were assigned DNA marker genotypes that were completely linked to a locus or loci associated with the trait of interest. Mean genotypic values of marker genotypes were derived based on the distribution of marker genotypes and the variance explained by marker(s).

Each population was assumed to consist of 400 single-clone individuals. *V*_P_ of both populations was set as 200 for convenience of calculations. Sixteen trait-test scenarios with unique combinations of *H* and *P* values were simulated, where each scenario was assigned an *H* value of 0.2, 0.5, 0.8 or 1.0 for the target trait and a *P* value of 0.2, 0.5, 0.8 or 1.0 for the DNA test. *V*_G_, *V*_E_, *V*_M_ and *V*_B_ were calculated based on *V*_P_, *H* and *P* for each scenario.

The first population involved three segregating genotypes that could be identified by a DNA test targeting a single trait locus with a completely linked marker. A quarter of the seedlings had the genotype MM, one half of the seedlings had genotype Mm and one quarter had genotype mm. The mean genotypic values of MM, Mm and mm were assumed to be *a*_3_, *d*_3_ and −*a*_3_, respectively, after being adjusted by subtracting the zero-point on the scale, which was assigned the value of 25. Because the value of the zero-point is independent of genetic gain, 25 was chosen so that the majority of seedlings had positive phenotypic values. For this segregation pattern, genetic gain was studied for cases of no dominance (*d*_3_=0), partial dominance (*d*_*3*_=*a*_*3*_/2) and complete dominance (*d*_3_=*a*_3_). The observed population mean (*μ*) was calculated as 25+*d*_3_/2 (Supplementary Equation S1). The value for *a*_3_ was calculated as $a_{3}=\sqrt{4\times V_{M}/(2+{\left(\frac{d_{3}}{a_{3}}\right)}^{2})}$ according to the distribution of marker genotypes (Table 1, Supplementary Equation S2).

The second population involved nine segregating genotypes identified by a DNA test targeting two unlinked trait loci: locus *M* with a major effect and locus *T* with a minor effect. Two markers composing this DNA test were completely linked to trait loci. Three possible genotypes were segregating for *M*: MM, Mm and mm, with genetic effects 2*a*_9_, *a*_9_ and −2*a*_9_, respectively. Three genotypes were segregating for *T*, where TT, Tt and tt had genetic effects *a*_9_, 0 and −*a*_9_, respectively. Epistasis was modeled between these two loci, where genotypes MMTt and mmTT had mean genotypic values of 3*a*_9_ (instead of 2*a*_9_) and −2.5*a*_9_ (instead of –*a*_9_), respectively. Frequency and mean genotypic values for nine segregating genotypes are shown in Table 2. The zero-point on this scale was assigned 25. The population mean equaled 25+17×*a*_9_/32 (Supplementary Equation S1). The value for *a*_9_ was calculated as $\sqrt{\frac{2}{5}\times V_{M}}$ (Table 1, Supplementary Equation S2).

### Analytical deduction of genetic gain

Genetic gains from *phenotype-only*, *marker-only*, two-stage and index were estimated using an analytical approach. Assuming that phenotype followed a normal distribution, genetic gain (∆*g*) from *phenotype-only* was derived as:

(6)Δgphenotype−only=H×iP×VP

where *i*_P_ was the selection intensity based on phenotypic information.

Genetic gain from *marker-only* was derived by subtracting the average marker effects before selection (*M*) from the average marker effects after selection (M'), assuming that marker effects were estimated without bias:

(7a)Δgmarker−only=M'−M

Under conditions where the distribution of marker genotypic values was approximately normal, genetic gain from *marker-only* could also be estimated as $i_{M}\sqrt{V_{M}}$ (7b), where *i*_M_ was the selection intensity based on marker information.

In two-stage, genetic gain in the first stage (Δ*g*_1_) was calculated according to Equation (7a), where $\Delta g_{1}=M'-M$, and genetic gain from the second stage (Δ*g*_2_) was calculated as:

(8)Δg2=H′×iP′×VP′

where $H^{'}$ was the broad-sense heritability after selection in the first stage, which was calculated as $H^{'}=(V_{M}^{'}+V_{B})/(V_{M}^{'}+V_{B}+V_{E})$, $V_{M}^{'}$ was the genotypic variance explained by marker loci after the first stage, iP' was the selection intensity after the first stage, and VP' was the phenotypic variance after the first stage. Combining genetic gains from two stages, the total genetic gain from two-stage was calculated as:

(9)Δgtwo−stage=Δg1+Δg2=M−M'+H'×iP'×VP'

Genetic gain from index selection, assuming the index followed a normal distribution, could be estimated by:

(10)Δgindex=H×iP×VP×P/H+(1−P)2/(1−H×P)

Equation 10 was modified from Lande and Thompson^15^ by replacing the narrow-sense heritability with *H*.

### Simulation of genotypic and phenotypic values

Genotypic and phenotypic values of each individual in a given scenario were simulated by first assigning a base value of 25 (the zero-point on the scale of assigned genotypic values) to each individual in the seedling population. Second, each individual was assigned a marker genotype according to the genotypic frequency distribution of the population, for example, in scenarios with three segregating genotypes, 100 seedlings were assigned genotype MM, 200 Mm and 100 mm. The marker effect (the mean genotypic value) was assigned based on an individual’s genotype. Third, the background genetic effect and environmental effect were randomly assigned to each individual from a normal distribution with mean 0 and variance *V*_B_ for the background genotypic effect and *V*_E_ for the environmental effect. The genotypic value of an individual was then calculated by summing the marker effect and background genetic effect (Equation 3). The phenotypic value of an individual was calculated by adding its environment effect to its genotypic value (Equation 2).

Selection was conducted based on simulated genotypic and phenotypic values. Genetic gain (Δ*g*) from alternative strategies was calculated as the increase in the mean genotypic value of selected individuals (g') compared with unselected individuals (*g*): $\Delta g=g^{'}-g$. Each simulation was repeated 1000 times, and the mean genetic gain and 95% confidence interval (CI) was calculated as:

(11)95%CI=x¯+1.96×sn

where $\overset{¯}{x}$ was the observed mean, *s* was the observed s.d. and *n* was the sample size (*n*=1000 in this study).

### Comparisons between derived and simulated results

In every trait-test scenario, derived mean genetic gain at every modeled TSP value was compared with simulated results at the same TSP. In two-stage seedling selection, comparisons were only made between mean genetic gains achieved at optimal SP_M_, for example, in the second scenario of the population with three segregating genotypes, optimal genetic gain was achieved when SP_M_ equaled 0.25 for a TSP equaled 0.1. Correlation coefficients between derived and simulated mean genetic gains at all TSP values were calculated in Excel 2007 to quantify closeness of derived and simulated results. For two-stage seedling selection, correlations were only calculated between optimal genetic gains based on derivation and simulation at every TSP values.

## Results

### Phenotypic distributions in 16 trait-test scenarios

**Figure 2:**
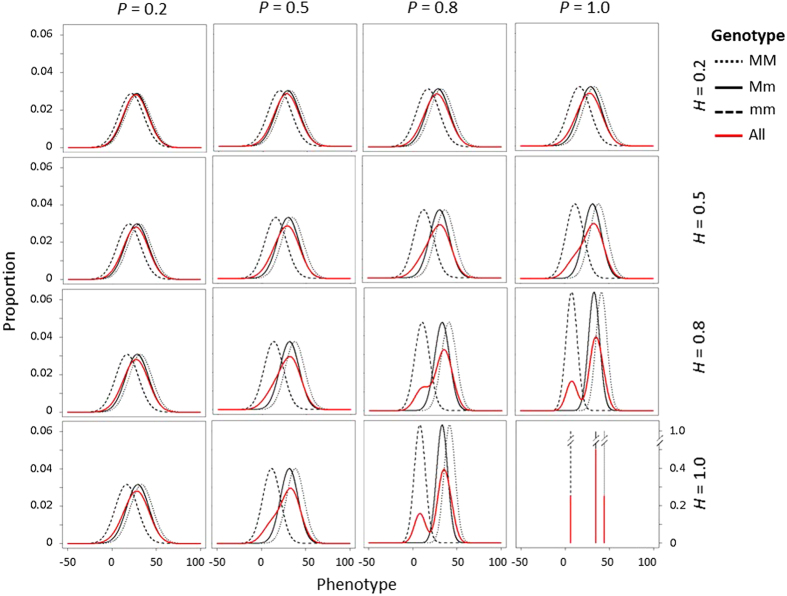
Phenotypic distributions in 16 scenarios for the population with three segregating genotypes and partial dominance (*d*_3_=*a*_3_/2). Black lines indicate phenotypic distributions of each single genotype, and red lines indicate phenotypic distributions of all seedlings in the population. Each graph represents phenotypic distributions of a scenario with a given broad-sense heritability (*H*) of the trait and predictiveness (*P*) of the DNA test. In each graph, the *X-*axis indicates phenotypic value and the *Y*-axis is the proportion of seedlings with a phenotypic value.

In the population with three segregating genotypes and partial dominance, the proportion of the total phenotypic variance explained by the marker locus (or loci) increased as *P* and *H* increased, which was indicated by greater differences between the mean phenotypic values of different genotypes (Figure 2). The phenotypic distributions of all seedlings deviated further from normal distributions as *P* and *H* increased (Figure 2). Multiple peaks were observed where *H* and *P* both reached 0.8. Where both *P* and *H* reached 1, phenotypic values of seedlings were arranged in discrete distributions where the phenotypic value of a seedling was determined only by its marker genotype. Similar patterns were also observed in the same population with zero or complete dominance and the population with nine segregating genotypes (Supplementary Figure. S1).

### Simulated and derived genetic gain in 16 trait-test scenarios

#### Genetic gain from *phenotype-only* seedling selection

**Figure 3:**
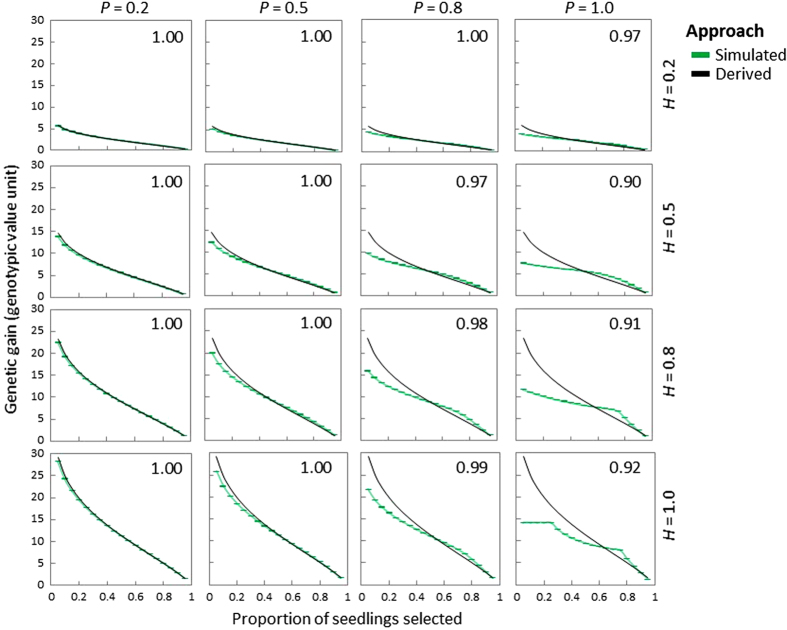
Comparison between derived and simulated genetic gains from phenotype-only seedling selection for the population with three segregating genotypes with partial dominance (*d*_3_=*a*_3_/2). Each plot represents a selection scenario with a given broad-sense heritability (*H*) of the trait and predictiveness (*P*) of the DNA test. In each plot, the *X-*axis indicates the proportion of seedlings selected in the end of seedling selection, ranging from 0.05 to 0.95. The *Y-*axis indicates genetic gain from seedling selection based on the unit of simulated genotypic values. Error bars for each data point indicate the 95% confidence interval (Equation 11), which are not obvious because of extremely tight confidence intervals. Numbers on the right corner of each plot are correlation coefficients between mean genetic gains estimated based on derivation and simulation.

In the population with three segregating genotypes and partial dominance, simulated genetic gain from *phenotype-only* decreased as TSP increased from 0.05 to 0.95 (Figure 3). The decrease in genetic gain followed a smooth curve in scenarios in which the phenotypic distribution was approximately normal, whereas where the normal distribution was violated, the decrease in genetic gain exhibited various patterns (Figure 3). For a constant value of TSP and *H*, simulated genetic gain tended to decrease with increasing *P*, and this was more pronounced at low values of TSP. Under the same TSP and *P*, simulated genetic gain increased as *H* increased from low to high (Figure 3). Derived and simulated genetic gains from *phenotype-only* were highly correlated in scenarios where the phenotypic distribution of the seedling population was approximately normal, whereas they were poorly correlated where the phenotypic distributions greatly deviated from normal distributions (Figure 3). For scenarios with similar phenotypic distributions (for example, Scenarios 12 and 15 in Figure 3), scenarios with high *H* values showed higher correlation coefficients between simulated and derived genetic gains compared with scenarios with low *H* values (Figure 3). Similar observations were also made in the same population where there was zero or complete dominance and in the population with nine segregating genotypes (Supplementary Figure S2).

#### Genetic gain from *marker-only* seedling selection

**Figure 4:**
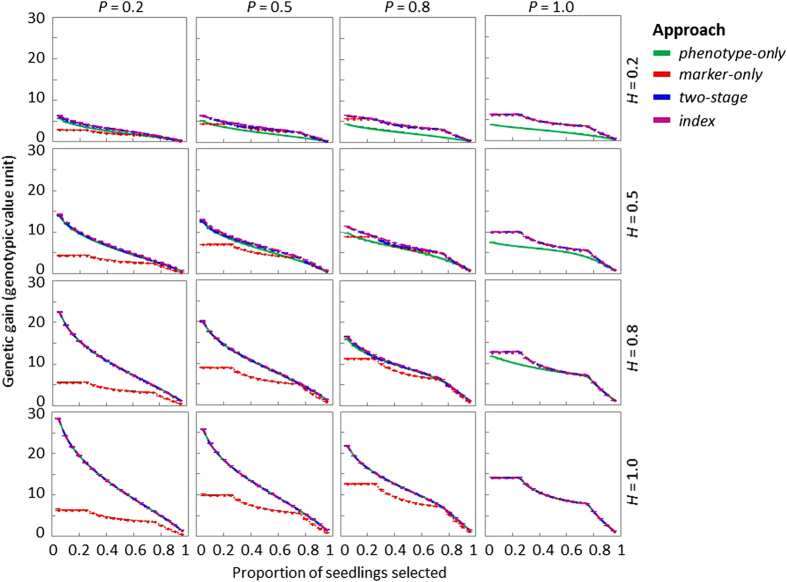
Simulated genetic gain from alternative seedling selection strategies for the population with three segregating genotypes and partial dominance (*d*_3_=*a*_3_/2). Each plot represents a selection scenario with a given broad-sense heritability (*H*) of the trait and predictiveness (*P*) of the DNA test. In each plot, the *X-*axis indicates the proportion of seedlings selected in the end of seedling selection, ranging from 0.05 to 0.95. The *Y-*axis indicates genetic gain from seedling selection based on the unit of simulated genotypic values. Error bars for each data point indicate the 95% confidence interval (Equation 11), which are not obvious because of extremely tight confidence intervals.

Optimal genetic gains based on derivation and simulation from *marker-only* matched closely in all scenarios and in all segregating populations (Supplementary Figure S3). In all populations, both simulated and derived genetic gain remained constant where TSP increased from 0.05 to the proportion of seedlings with the best marker genotype, for example, 0.25 for the population with three segregating genotypes and zero or partial dominance (Figure 4 and Supplementary Figure S3b). Genetic gain decreased as TSP increased to 0.95. The decrease of genetic gain from *marker-only* followed a smoother curve in the population with nine segregating genotypes compared with populations with three segregating genotypes (Supplementary Figure S3d). In all populations, where *H* and TSP remained constant, genetic gain increased as *P* increased; where *P* and TSP remained constant, increases in genetic gain were also observed as *H* increased. Genetic gain reached the highest values where both *P* and *H* were at the extreme value of 1, where all phenotypic variance was attributed to the marker locus/loci.

#### Genetic gain from two-stage seedling selection

Similar to the results in *marker-only* selection, simulated genetic gain from two-stage decreased as TSP increased to 0.95 (Figure 4 and Supplementary Figure S4). The decrease in genetic gain tended to follow a similar pattern as *phenotype-only* where *H* was greater than *P*, whereas the pattern was more similar to *marker-only* where *H* was less than *P*. Derived genetic gains from two-stage were highly correlated with simulated genetic gains in most trait-test scenarios for the majority of the populations except for scenarios in which the phenotypic distribution in the second stage was far from normal and genetic gain from the second stage was on the same scale as that from the first stage, especially at low TSP values (Supplementary Figure S4).

#### Genetic gain from index seedling selection

Simulated genetic gain from index followed a similar pattern as two-stage, which achieved similar genetic gain as *phenotype-only* where *H* was greater than *P*, whereas index was equivalent to *marker-only* where *H* was less than *P* (Figure 4 and Supplementary Figure S5). Derived results tended to more closely match simulated results where the ratios between weight coefficients of marker score and phenotypic score (*b*_*m*_/*b*_*z*_) were low and the phenotypic distributions were close to normal (Supplementary Figure S5).

### Comparison of simulated genetic gain among four alternative seedling selection strategies

Comparing populations with various genetic structures, the pattern of genetic gain changing with increasing TSP was influenced by the number of segregating genotypes and the degrees of dominance and epistasis (Figure 4 and Supplementary Figure S6). Despite different patterns in genetic gain changes, two-stage and index seedling selection were always associated with similar genetic gain and both achieved as high, or higher, genetic gain as the best of *phenotype-only* and *marker-only* in all populations and scenarios (Figure 4 and Supplementary Figure S6). Genetic gains achieved from two-stage and index were similar to that from *marker-only* seedling selection where *P* was much greater than *H* and *phenotype-only* where *H* was much greater than *P* (Figure 4 and Supplementary Figure S6). In all populations evaluated, genetic gain from *marker-only* tended to be greater than that from *phenotype-only* where *P* was greater than *H*, and less where *P* was less than *H* (Figure 4 and Supplementary Figure S6). Where *P* equaled *H*, genetic gain from *marker-only* was similar to that from *phenotype-only*. In all scenarios, highest relative genetic gain from *marker-only* over *phenotype-only* was likely to be achieved where all seedlings with favorable marker genotypes were selected and no random selection was made in any marker genotype, especially where *P* was low and a few (for example, three) genotypes were segregating in the seedling population (Figure 4 and Supplementary Figure S6). Relative genetic gain from *marker-only* compared with *phenotype-only* tended to be optimized at a few TSP values where no random selection was made.

### Influence of the proportion of seedlings selected in the first stage on genetic gain from two-stage seedling selection

**Figure 5:**
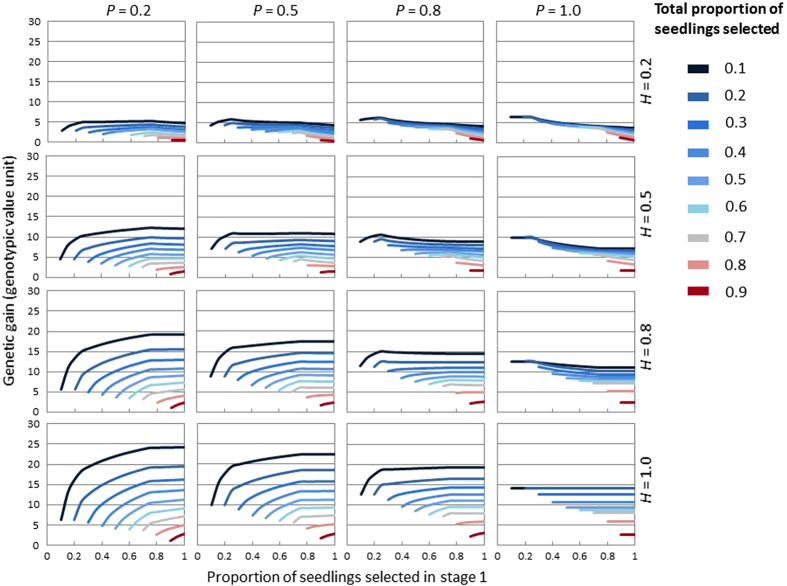
Simulated genetic gain from two-stage seedling selection for the population with three segregating genotypes and partial dominance (*d*_3_=*a*_3_/2). Each plot represents a selection scenario with a given broad-sense heritability (*H*) of the trait and predictiveness (*P*) of the DNA test. In each plot, the *X*-axis indicates the proportion of seedlings selected in the first stage, and the *Y*-axis indicates simulated genetic gain from two-stage seedling selection based on SP_M_.

In all scenarios, genetic gain from two-stage seedling selection at any SP_M_ tended to decrease as TSP increased (Figure 5 and Supplementary Figure S7). Where *P* was greater than or equal to *H*, the highest genetic gain was achieved where as many as seedlings as possible were selected based on marker information (Figure 5 and Supplementary Figure S7). Where *P* was less than *H*, relying on *phenotype-only* or discarding only seedlings with the most undesirable genotype in the first stage of two-stage was associated with higher genetic gain. Where both *P* and *H* equaled 1, changes in the proportion of seedlings selected in the first stage did not influence genetic gain and two-stage generated the same genetic gain as *marker-only* and *phenotype-only*.

## Discussion

This study modeled genetic gain from four alternative seedling selection strategies on a single trait basis through using analytical derivation and stochastic simulation on a generalized seedling selection scheme for clonally propagated crops. Guidelines were proposed for optimizing genetic gain as well as the overall efficiency from seedling selection for single traits. Discussion was further extended to choosing selection strategies for multiple traits to optimize the overall selection efficiency in terms of genetic gain, cost and time.

### Comparison between analytical derivation and stochastic simulation

Comparisons between derived and simulated results indicate that the accuracy of analytical derivation is restricted by the fulfillment of assumptions embedded in equations for calculating genetic gains (Equations 6–10). Estimated mean genetic gain from *phenotype-only* based on Equation (6) was poorly correlated with simulated results where the assumption of normal distribution were violated (Figure 3 and Supplementary Figure S2). High correlation coefficients between derived and simulated genetic gains from *marker-only* seedling selection (Supplementary Figure S3) were due to assumptions made in simulation, such as no bias in estimated marker effects, markers completely linked to trait loci, and/or normally distributed background genotypic and environmental effects. Predicted genetic gain from two-stage seedling selection tended to be less accurate where the phenotypic distribution in the second stage was far from being normal and where genetic gain from the second stage was similar to or higher than that from the first stage (Supplementary Figure S4). In some scenarios, low correlations between derived and simulated genetic gains from index seedling selection (Supplementary Figure S5) were likely caused by non-normal distributions of the selection index (Equation 10), where either the distribution of the phenotypic score (for example, both *P* and *H* were both great) or marker score (for example, there were very few discrete marker scores) was far from normal, and significant weight was put on the non-normally distributed parameter(s). Equations used in previous studies were also restricted by assumptions such as normal distribution of phenotypic values or selection index.^15,24^ The accuracy and flexibility of analytical derivation in predicting genetic gain would be further improved by deriving equations suitable for various phenotypic and genotypic distributions.

### Comparison of genetic gain among alternative seedling selection strategies

Relationships between relative genetic gain from *marker*-*only* over *phenotype*-*only* and the ratio between *P* and *H*, as observed in simulation results (Figure 4 and Supplementary Figure S6), was supported by analytical derivation. Relative genetic gain from *marker*-*only* compared to *phenotype*-*only* seedling selection is estimated as $\frac{i_{M}}{i_{P}}\times \sqrt{P/H}$ (Equation 6, 7b). If *i*_*M*_ roughly equals *i*_*P*_, *marker*-*only* tends to generate higher genetic gain compared to *phenotype*-*only* seedling selection where *P* was greater than *H*, and vice versa. Similar conclusions were made by Smith^24^ based on an animal breeding model in which only additive variances were considered. Instead of using *P* and *H*, the relative efficiency of *marker*-*only* selection compared to phenotypic selection in additive models depends on the proportion of the total additive genetic variance due to the known loci (*p*) relative to the narrow-sense heritability of the trait (*h*^*2*^). The use of *P* and *H* in this study reflects the unique feature of seedling selection for clonally propagated crops, where the total genotypic effects are captured during clonal propagation.^2^ The ratio between estimated *P* and *H* can thus serve as an indicator of relative genetic gain from *marker*-*only* over *phenotype*-*only* for clonally propagated crops under the same selection intensity.

Random selection made in *marker*-*only* to meet a given *TSP* tended to sacrifice genetic gain from seedling selection especially where *P* was low and a few genotypes were segregating in the seedling population. This impact of random selection on genetic gain from MASS was because those individuals discarded randomly might have higher genotypic values compared to the selected ones at low *P*-values. Most previous studies either focused on *marker*-*only* without random selection^28^ or in seedling populations with many segregating genotypes,^27^ in which cases the influence of random selection on genetic gain was not obvious. Based on results in this study, limiting the amount of random selection during *marker*-*only* seedling selection tends to achieve higher genetic gain where *P* is low and only a few marker genotypes are segregating.

*Two*-*stage* and *index* seedling selection tended to optimize genetic gains compared to *phenotype*-*only* and *marker*-*only* seedling selection (Figure 4 and Supplementary Figure S6) because *two*-*stage* and *index* seedling selection take advantage of both phenotypic and genotypic information by weighting them optimally. Genetic gains achieved from *two*-*stage* and *index* were similar to *marker*-*only* seedling selection where *P* was much greater than *H* and *phenotype*-*only* where *H* was much greater than *P* (Figure 4 and Supplementary Figure S6), indicating that additional information provided by combining phenotypic and genotypic information did little to increase accuracy of predicting genotypic values if one type of information was much more predictive than the other. Thus, the use of *two*-*stage* and *index* is more likely to increase genetic gain compared to *phenotype*-*only* or *marker*-*only* seedling selection where phenotypic and genotypic information can complement each other to generate optimal genetic gains. Similar findings were reported in additive models, where *h*^*2*^ and *p* were studied instead of *H* and *P*.^23,15^ Studies by Hospital et al.^25^ and Moreau et al.^29^ considered factors influencing the accuracy of predicted marker effects and suggested that the efficiency of index selection is reduced by the low power of trait locus detection in populations of finite size especially if heritability is lower than 0.2. Although assumed to be known in this study, in reality the exact marker effects are often estimated with error. Therefore, considering the accuracy of predicted marker effects is important for choosing efficient selection strategies: marker-based strategies are genetically more efficient than *phenotype*-*only* seedling selection if they can achieve higher genetic gain regardless of imperfect estimation of marker effects.

Slightly higher genetic gains from *index* compared to *two*-*stage* seedling selection in some circumstances (e.g., Scenario 7 in Supplementary Figure. S6c) is likely caused by *index* taking into account both phenotypic and genotypic information simultaneously while *two*-*stage* did so separately. Seedlings with highest genotypic values might have been discarded in the first stage if marker scores do not reflect the true genotypic potential, especially at low *P* values. Similar observations were made in sexually propagated crops.^15,28,30^ Considering small differences between optimal genetic gains achievable from *index* and *two*-*stage* (Figure 4 and Supplementary Figure S6), time and cost of *index* and *two*-*stage* seedling selection play more important roles in determining efficiency of these two strategies.

The influence of the proportion of seedlings selected in the first stage (*SP*_*M*_) on genetic gains from *two*-*stage* seedling selection (Figure 5 and Supplementary Figure S7) indicate that optimal genetic gain from *two*-*stage* is achieved when genotypic and phenotypic information is optimally weighted. Where *P* was greater than *H*, selecting against the most undesirable marker genotype in the first stage tended to achieve the optimal genetic gain because that marker information was more accurate in predicting genotypic values than was phenotypic information. In contrast, where *H* and *P* were similar, the value of *SP*_*M*_ had less effect on genetic gain from *two*-*stag*e seedling selection because phenotypic and genotypic information was equally predictive. Methods of choosing selection proportions in the first stage to optimize genetic gain have been reported for multiple-trait selection,^31,32^ but no study has reported results for single traits. This study simulated genetic gains from *two*-*stage* seedling selection by choosing all possible thresholds in the first phase. In future, theoretical studies are needed to provide easier ways to determine the selection threshold in the first phase to optimize genetic gain from *two*-*stage* seedling selection.

It is impossible to model populations with all possible segregating patterns; however, a similar trend observed of relative genetic gain from marker-based strategies over *phenotype-only* seedling selection in all populations modeled in this study, supported by theoretical derivation, suggested that the ratio between predictiveness of the DNA test and broad-sense heritability of the trait can be used as a general indicator for choosing strategies with optimal genetic gain, regardless of numbers of marker loci or segregating genotypes involved. Similarly, previous studies based on additive models suggested that indication of the ratio between proportion of additive variance explained by markers and narrow-sense heritability on relative genetic gain from marker-assisted selection over traditional selection was not restricted by the number of marker loci or segregating genotypes involved.^15,24^

### Limitations and future work

Some assumptions made in this study might not be met when practically deploying MASS in a breeding program. The assumption that the exact effects of trait loci and values of genetic parameters such as *H* and *P* were known and that alleles on those trait loci could be perfectly determined by markers is often not met in reality. The accuracy of estimated marker effects is influenced by many variable factors such as the size of the population on which the estimation was made, its genetic relationship to the breeding germplasm targeted for MASS deployment, and the extent to which linkage phase relationships between alleles of markers and trait loci are maintained between the estimation population and the target population.^25,29,^^33^ Accurate estimation of *H* and *P* values also depends on the use of statistical models that capture the total genotypic variance. In practice, if additive genetic action only is modeled, estimated relative genetic gain from marker-based strategies over *phenotype-only* seedling selection might be biased especially when non-additive effects are substantial, as observed in the results for simulated populations with included dominance and epistasis gene actions (Supplementary Figure S6). Further assumptions in this study also assumed no interaction between alleles at marker loci and alleles in background genomes, and normally distributed environmental variance. If traits under selection do not meet those assumptions, realized genetic gain from marker-based strategies with relatively high predicted genetic gain might not exceed that from *phenotype-only* seedling selection.

In situations where assumptions do not match with reality, deploying MASS requires extra caution, but directed efforts could improve predictions. Effective deployment of MASS would benefit from studies that: (1) increase *P* by identifying markers associated with additional loci for the trait under selection and incorporating them into the DNA test, and (2) increase the accuracy of estimated marker effects by using populations closely related to target breeding germplasm and adopting statistical models that capture the total genotypic variance. Also, the accuracy of predicted genetic gain could be improved by using more sophisticated models that account for factors such as errors in estimated genetic parameters (for example, marker effects, *H* and *P*-values), recombination probability between marker and trait loci, interactions between marker loci and background genomes and non-normally distributed environmental variance.

For multiple trait selection, the general outcomes of this study remain relevant, particularly for independent selection thresholds, but further research is required. Rather than selecting single traits, breeders often focus on multiple traits during seedling selection.^30^ Studies on the genetic gain of marker-assisted (seedling) selection for multiple traits have been conducted for seed-propagated crops^30^ but not for clonally propagated crops. Selection thresholds delimiting attributes that are valuable in new cultivar candidates but not absolutely required (such as exceptional sweetness or very long storability) should be applied simultaneously with those for other traits by weighting according to the breeding priorities and considering genetic correlations among traits.^30^ However, selection thresholds delimiting attributes that are required for viable new cultivar candidates and that do not affect probabilities of seedlings achieving other required thresholds (that is, traits not genetically correlated) should be able to be applied independently following principles described above for single traits. Any given breeding program is likely to have numerous such selection thresholds (for example, for apple, a certain minimum fruit size, sweetness level and yield). Identifying strategies with optimal genetic gain for enhancing multiple selection thresholds requires a sophisticated framework that considers all selection thresholds simultaneously^15,30^ or in multiple stages.^30–32^ Further development of concepts and methods for determining genetically efficient MASS schemes for multiple traits in breeding of clonally propagated crops would facilitate effective MASS, particularly where genetic correlations are expected and for the many non-essential selection thresholds that breeding programs typically deal with.

In addition to genetic gain, the influences of cost and time on overall efficiency of seedling selection need to be considered. As pointed by Ru *et al.*,^6^ a major challenge of choosing efficient selection strategies is a lack of methods for quantifying and comparing selection efficiency of alternative strategies by weighting genetic gain, cost and time based on the breeding program’s needs. The utility for clonally propagated crops of units of overall breeding efficiency used in previous studies such as genetic gain per unit cost, genetic gain per unit time and cost per unit time^10,18,22,25^ will likely vary with breeding circumstances. Genetic gain per generation or cost per generation is not as informative in seedling selection as they are in recurrent selection where multiple generations are involved. Development of new units might also be useful for weighting the three parameters of selection efficiency to better fulfill a breeding program’s needs. Empirical evaluations of realized genetic gain and the overall efficiency of MASS could be used to validate conclusions from analytical and simulation studies and improve current models. Moreover, investigations of the overall efficiency of the whole selection process, including selection phases after seedling selection, would facilitate efficient selection beyond the scope of seedling selection.

## Figures and Tables

**Table 1:** Trait-test scenarios and derived additive effects

*Scenario*	*Assigned parameters*			*Derivation*							
	H	P	V_*P*_	V_*G*_	V_*E*_	V_*M*_	V_*B*_	a_*3*_			a_*9*_
								d_*3*_*=0*	d_*3*_*=*a_*3*_*/2*	d_*3*_*=*a_*3*_	
1	0.2	0.2	200	40	160	8	32	4	3.8	3.3	1.5
2	0.2	0.5	200	40	160	20	20	6.3	6	5.2	2.3
3	0.2	0.8	200	40	160	32	8	8	7.5	6.5	3
4	0.2	1	200	40	160	40	0	8.9	8.4	7.3	3.3
5	0.5	0.2	200	100	100	20	80	6.3	6	5.2	2.3
6	0.5	0.5	200	100	100	50	50	10	9.4	8.2	3.7
7	0.5	0.8	200	100	100	80	20	12.7	11.9	10.3	4.7
8	0.5	1	200	100	100	100	0	14.1	13.3	11.6	5.2
9	0.8	0.2	200	160	40	32	128	8	7.5	6.5	3
10	0.8	0.5	200	160	40	80	80	12.7	11.9	10.3	4.7
11	0.8	0.8	200	160	40	128	32	16	15.1	13.1	5.9
12	0.8	1	200	160	40	160	0	17.9	16.9	14.6	6.6
13	1	0.2	200	200	0	40	160	8.9	8.4	7.3	3.3
14	1	0.5	200	200	0	100	100	14.1	13.3	11.6	5.2
15	1	0.8	200	200	0	160	40	17.9	16.9	14.6	6.6
16	1	1	200	200	0	200	0	20	18.9	16.3	7.4

*a*_3_ and *a*_9_ indicate additive effects for populations with three and nine segregating genotypes, respectively.

**Table 2:** Mean genotypic value and frequency for the population with nine segregating genotypes

	*Genotype*								
	*MMTT*	*MMTt*	*MMtt*	*MmTT*	*MmTt*	*Mmtt*	*mmTT*	*mmTt*	*mmtt*
Frequency	1/16	2/16	1/16	2/16	4/16	2/16	1/16	2/16	1/16
Mean genotypic value	3*a*_9_	3*a*_9_	*a*_9_	2*a*_9_	*a*_9_	0	−2.5*a*_9_	−2*a*_9_	−3*a*_9_
