# Supplementary material for: Modeling of genetic gain for single traits from marker-assisted seedling selection in clonally propagated crops
Source: Hortic Res. 2016 Apr 20;3:16015–. doi: 10.1038/hortres.2016.15 (PMC4837533; doi:10.1038/hortres.2016.15)
Supplement: Supplementary Information [file hortres201615-s1.pdf]

Supplementary materials

$$\mu = \sum_{i=1}^{n} g_{Mi} \times p_{Mi} \qquad \text{(S1)}$$

where μ was the mean phenotypic or genotypic value of the seedling population, n was the number of marker genotypes segregating in the seedling population, g_{Mi} was the marker genotypic value of the *i*th marker genotype, p_{Mi} was the frequency of the *i*th marker genotype.

$$V_M = \sum_{i=1}^{n} (g_{Mi} - \mu)^2 \times p_M \qquad \text{(S2)}$$

where V_M was the variance explained by marker loci, μ was the mean phenotypic or genotypic value of the seedling population, n was the number of marker genotypes segregating in the seedling population, g_{Mi} was the marker genotypic value of the *i*th marker genotype, p_{Mi} was the frequency of the *i*th marker genotype.

Table S1. Abbreviations of terminologies

Abbreviation	Definition
CI	95% confidence interval
e	environmental effect
g	genotypic value
g_M	effect of trait loci explained by the markers in the DNA test
g_B	background genotypic effect caused by genotypes at all influencing loci in the genome other than the DNA test-targeted loci
H	broad-sense heritability
h^2	narrow-sense heritability
index	*index* seedling selection
i_P	selection intensity based on phenotypic information
M	average marker effects
MASS	marker-assisted seedling selection
marker-only	*marker-only* seedling selection
P	predictiveness of the DNA test, which was calculated as the proportion of genotypic variance explained by marker loci used in the DNA test
p	proportion of the total additive genetic variance caused by the known loci
phenotype-only	*phenotype-only* seedling selection
two-stage	*two-stage* seedling selection
V_B	variance explained by the background genotypic effect caused by genotypes at all influencing loci in the genome other than the DNA test-targeted loci
V_E	environmental variance
V_G	genotypic variance
V_M	variance explained by markers
V_P	phenotypic variance
z	phenotypic value of an individual
Δg	genetic gain
Δg_1	genetic gain in the first stage of two-stage seedling selection
Δg_2	genetic gain in the second stage of two-stage seedling selection

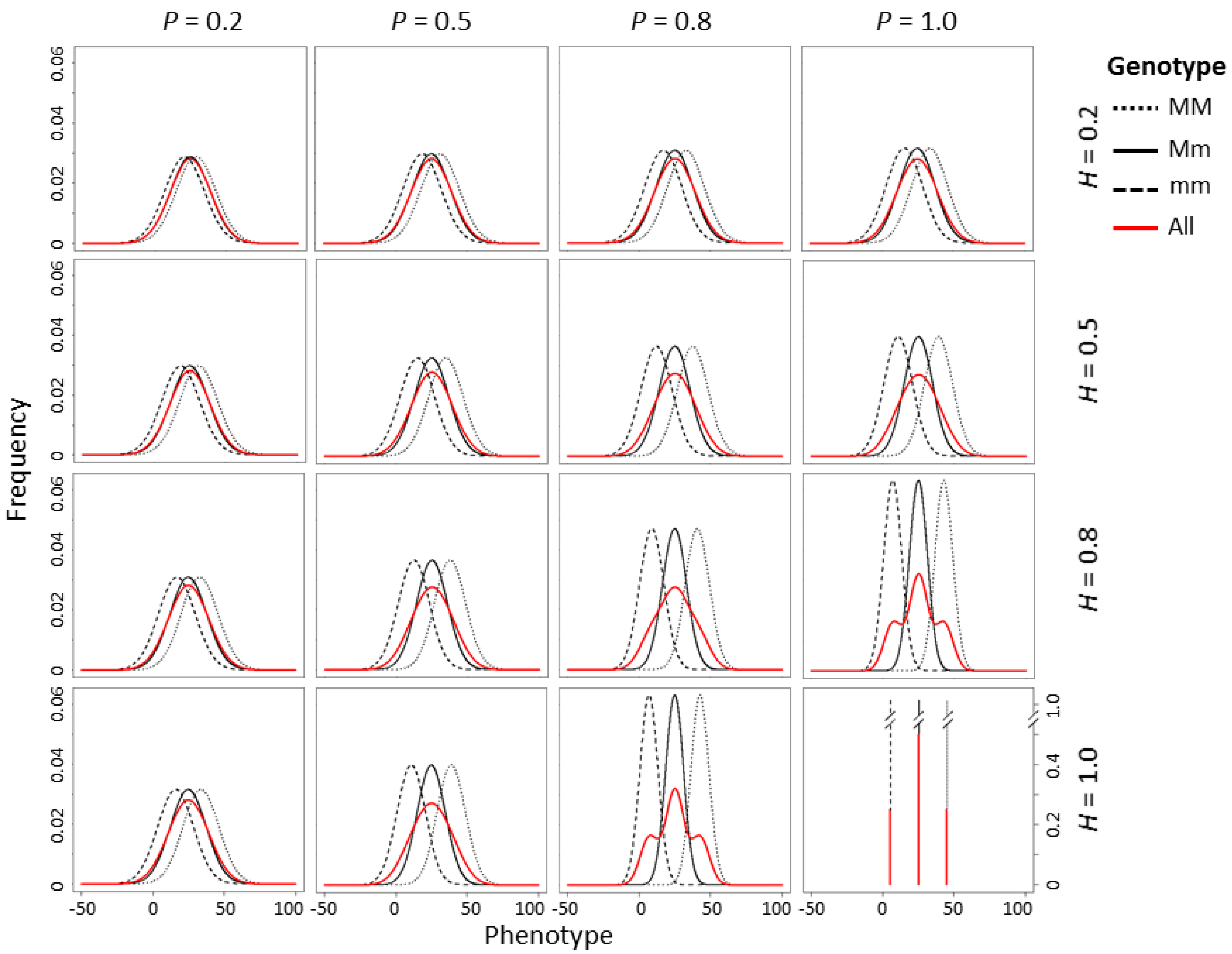

Fig. S1 (a) Phenotypic distributions in sixteen scenarios for the population with three segregating genotypes and no dominance ($d_3 = 0$)

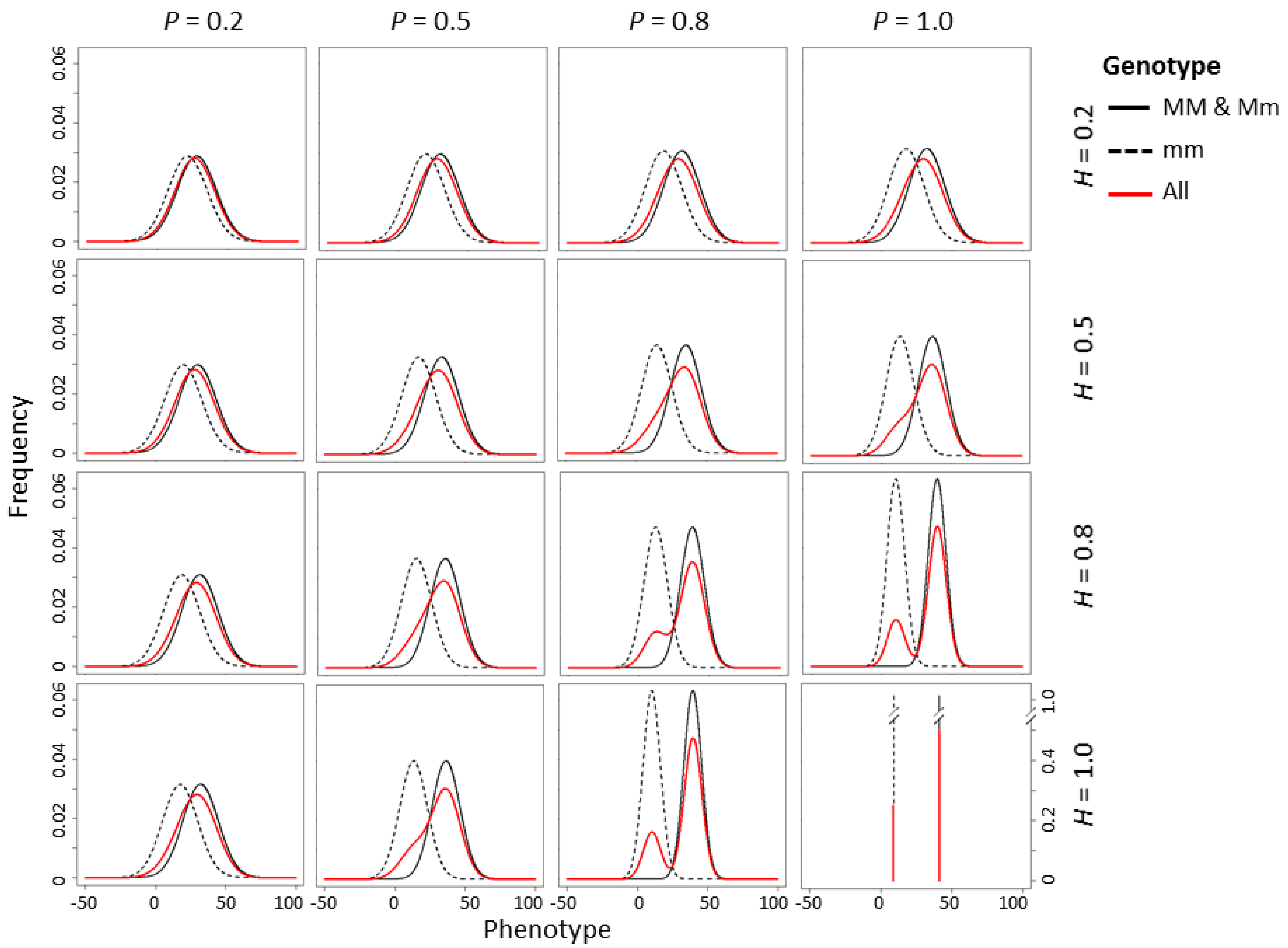

Fig. S1 (b) Phenotypic distributions in sixteen scenarios for the population with three segregating genotypes and complete dominance ($d_3 = a_3$)

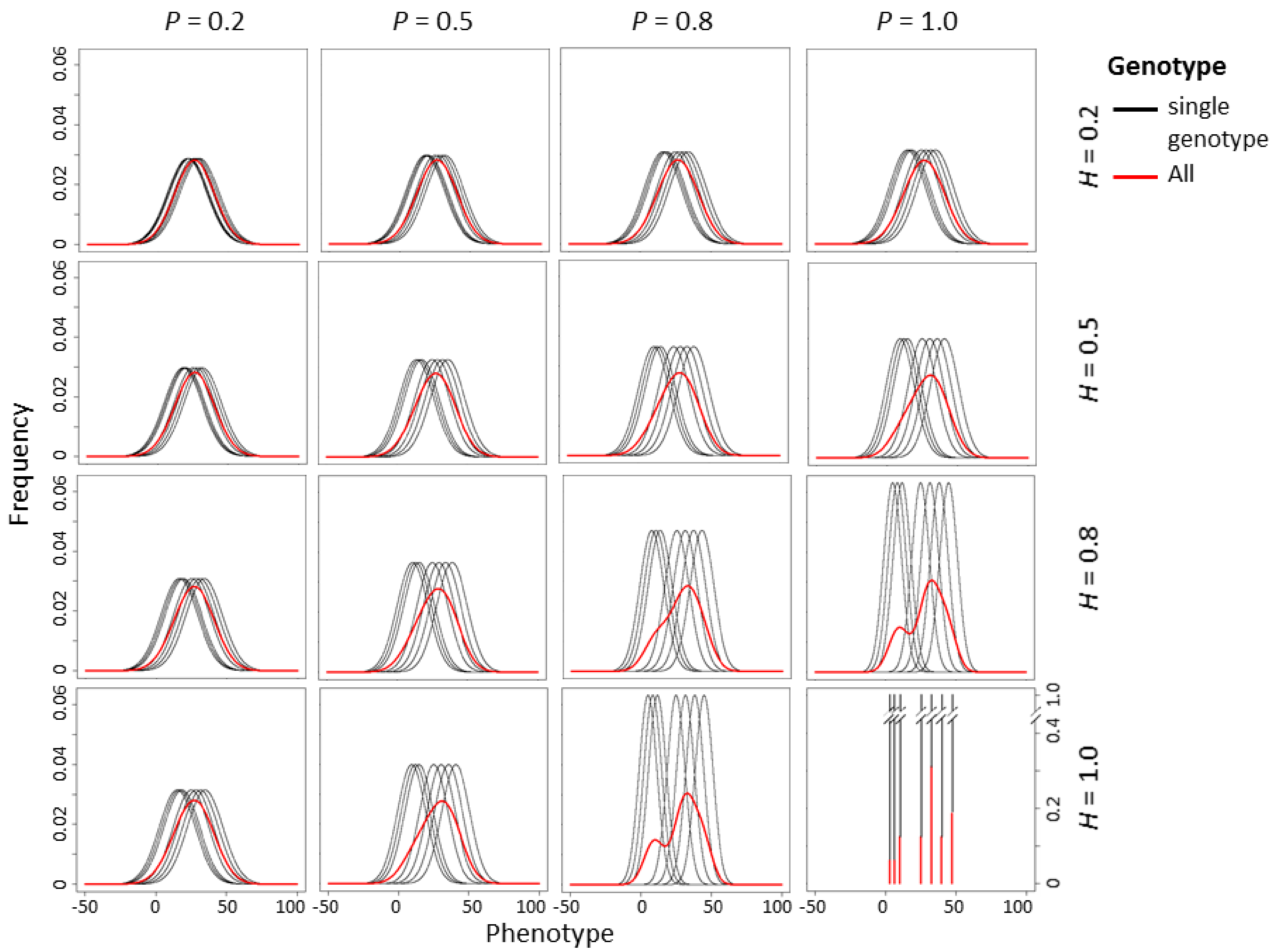

Fig. S1 (c) Phenotypic distributions in sixteen scenarios for the population with nine segregating genotypes

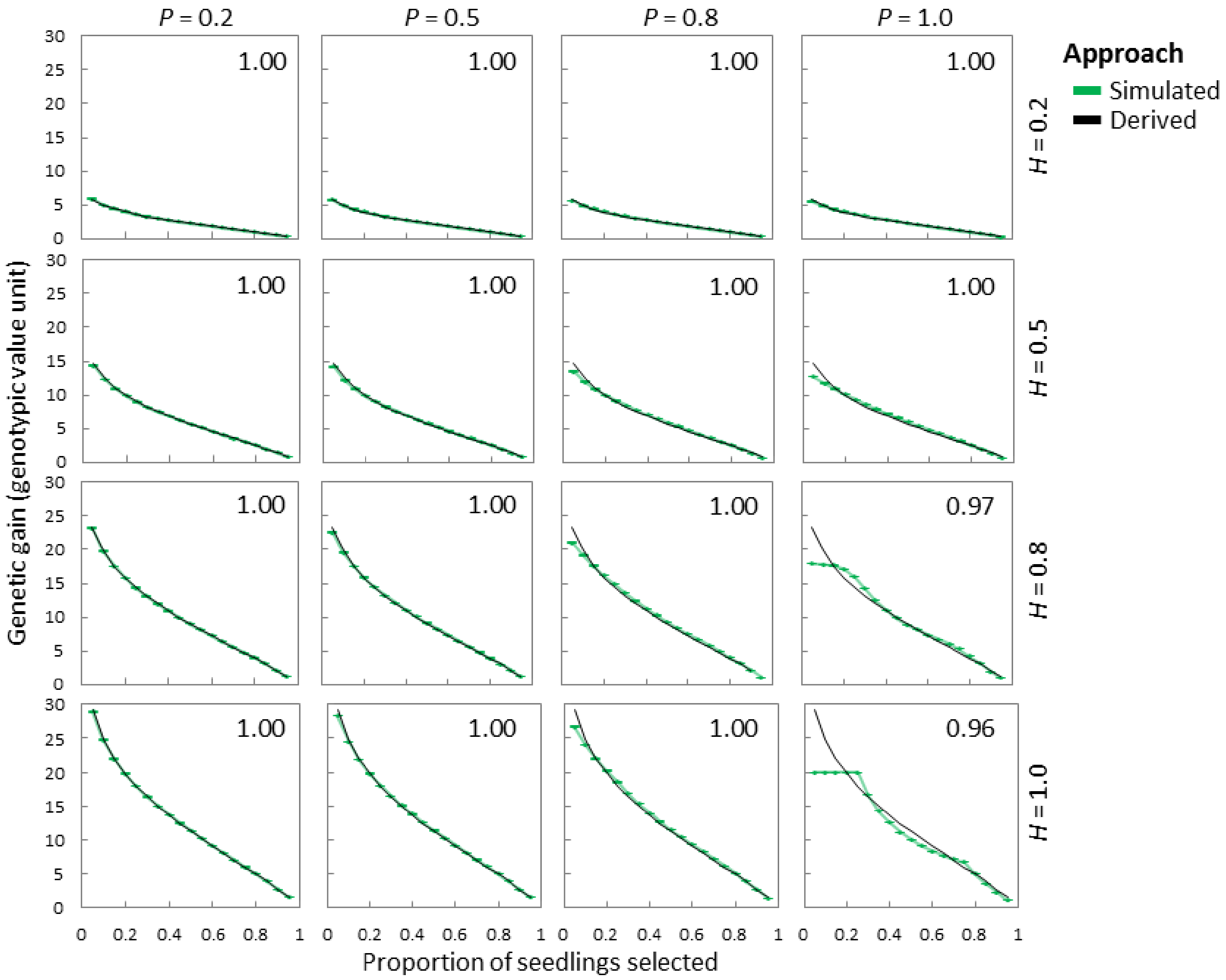

Fig. S2 (a) Comparison between derived and simulated genetic gains from *phenotype-only* seedling selection for the population with three segregating genotypes without dominance (d_3 = 0). Each plot represents a selection scenario with a given broad-sense heritability (*H*) of the trait and predictiveness (*P*) of the DNA test. In each plot, the X axis indicates the proportion of seedlings selected in the end of seedling selection, ranging from 0.05 to 0.95. The Y axis indicates genetic gain from seedling selection based on the unit of simulated genotypic values. Error bars for each data point indicate the 95% confidence interval (**Equation 11**), which are not obvious because of extremely tight confidence intervals. Numbers on the right corner of each plot are correlation coefficients between mean genetic gains estimated based on derivation and simulation.

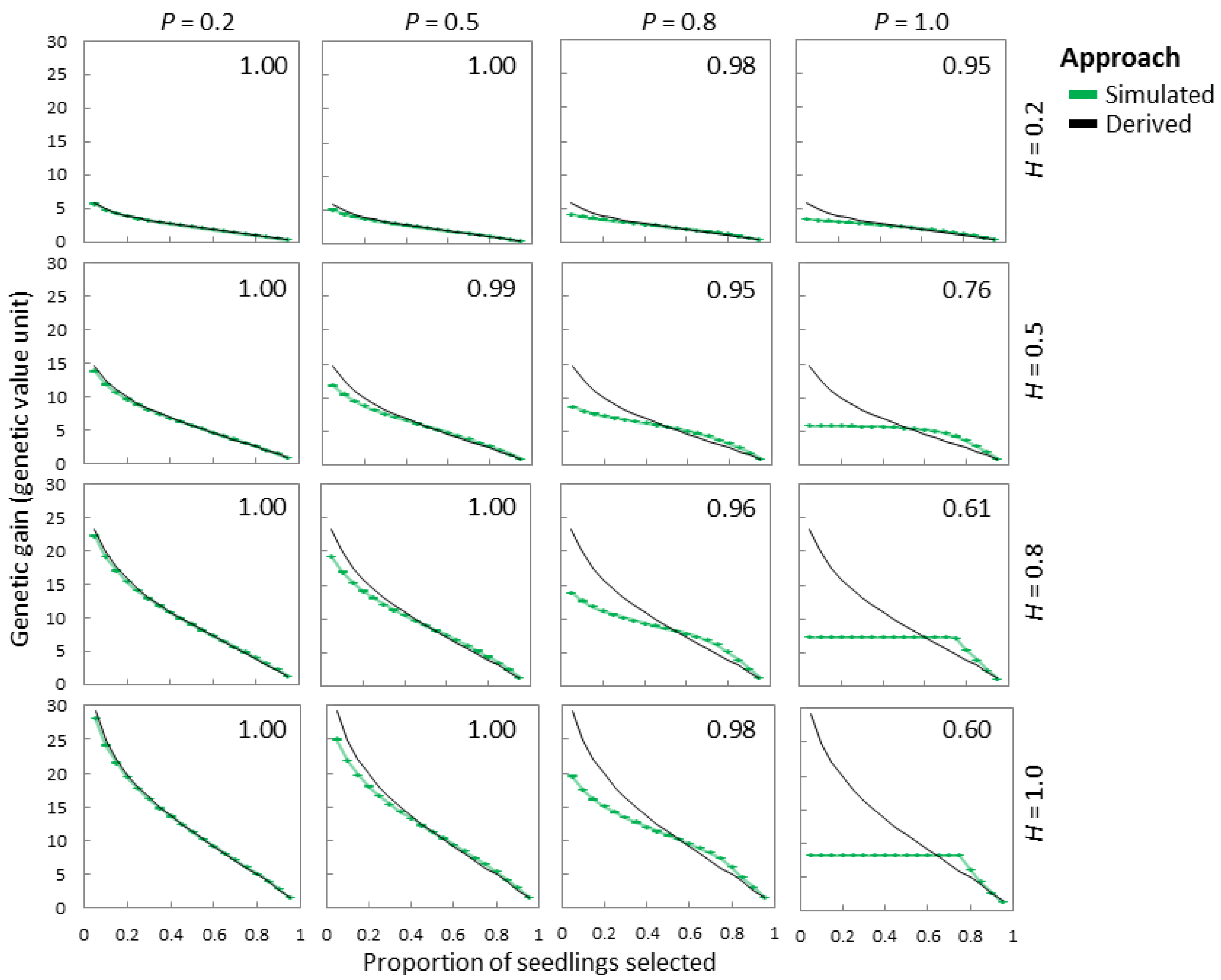

Fig. S2 (b) Comparison between derived and simulated genetic gains from *phenotype-only* seedling selection for the population with three segregating genotypes with complete dominance (d_3 = a_3). Each plot represents a selection scenario with a given broad-sense heritability (*H*) of the trait and predictiveness (*P*) of the DNA test. In each plot, the X axis indicates the proportion of seedlings selected in the end of seedling selection, ranging from 0.05 to 0.95. The Y axis indicates genetic gain from seedling selection based on the unit of simulated genotypic values. Error bars for each data point indicate the 95% confidence interval (**Equation 11**), which are not obvious because of extremely tight confidence intervals. Numbers on the right corner of each plot are correlation coefficients between mean genetic gains estimated based on derivation and simulation.

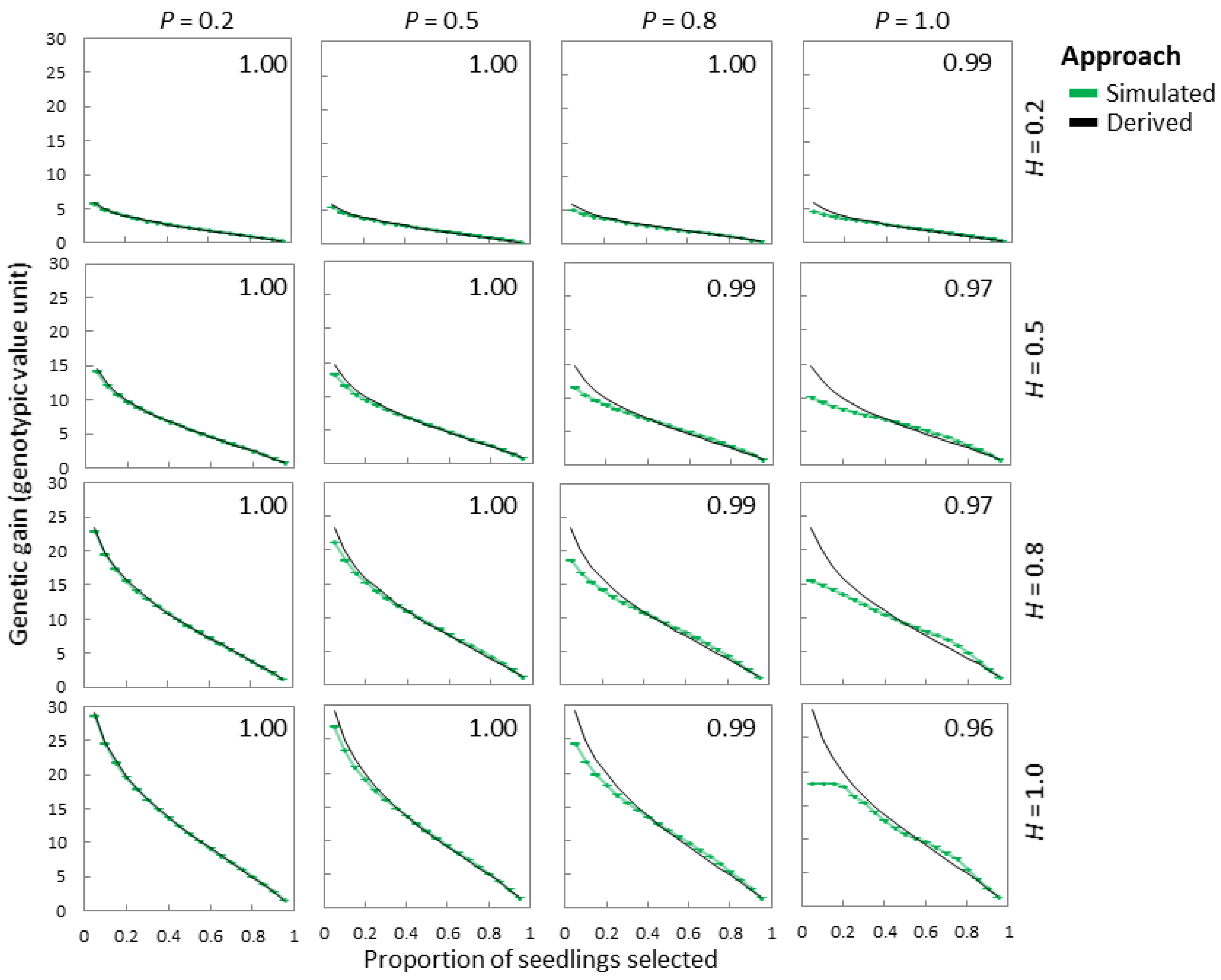

Fig. S2 (c) Comparison between derived and simulated genetic gains from *phenotype-only* seedling selection for the population with nine segregating genotypes. Each plot represents a selection scenario with a given broad-sense heritability (*H*) of the trait and predictiveness (*P*) of the DNA test. In each plot, the X axis indicates the proportion of seedlings selected in the end of seedling selection, ranging from 0.05 to 0.95. The Y axis indicates genetic gain from seedling selection based on the unit of simulated genotypic values. Error bars for each data point indicate the 95% confidence interval (**Equation 11**), which are not obvious because of extremely tight confidence intervals. Numbers on the right corner of each plot are correlation coefficients between mean genetic gains estimated based on derivation and simulation.

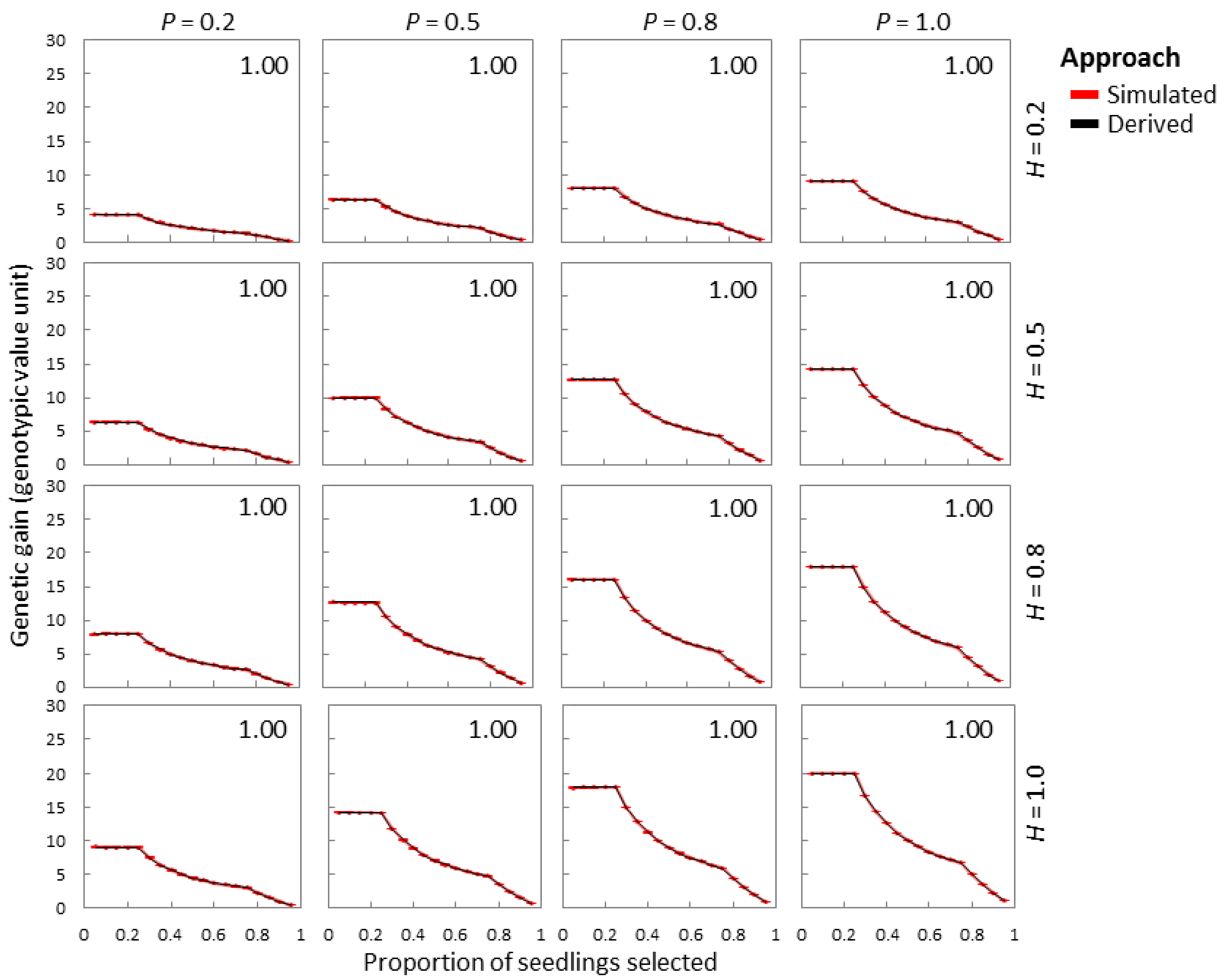

Fig. S3 (a) Comparison between derived and simulated optimal genetic gains from *marker -only* seedling selection for the population with three segregating genotypes without dominance (d_3 = 0). Each plot represents a selection scenario with a given broad-sense heritability (*H*) of the trait and predictiveness (*P*) of the DNA test. In each plot, the X axis indicates the proportion of seedlings selected in the end of seedling selection, ranging from 0.05 to 0.95. The Y axis indicates genetic gain from seedling selection based on the unit of simulated genotypic values. Error bars for each data point indicate the 95% confidence interval (**Equation 11**), which are not obvious because of extremely tight confidence intervals. Numbers on the right corner of each plot are correlation coefficients between mean genetic gains estimated based on derivation and simulation.

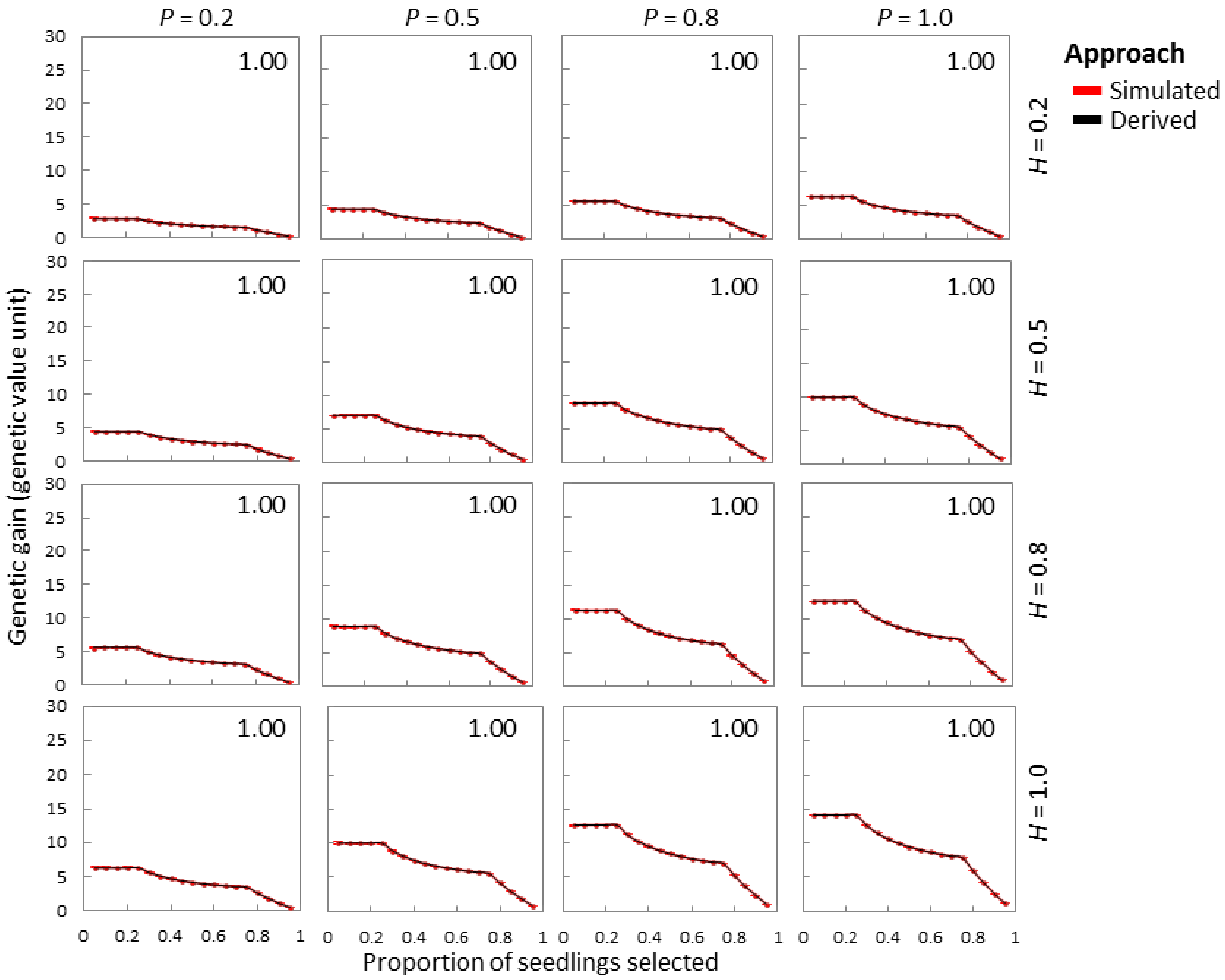

Fig. S3 (b) Comparison between derived and simulated optimal genetic gains from *marker - only* seedling selection for the population with three segregating genotypes with partial dominance (d_3 = a_3/2). Each plot represents a selection scenario with a given broad-sense heritability (*H*) of the trait and predictiveness (*P*) of the DNA test. In each plot, the X axis indicates the proportion of seedlings selected in the end of seedling selection, ranging from 0.05 to 0.95. The Y axis indicates genetic gain from seedling selection based on the unit of simulated genotypic values. Error bars for each data point indicate the 95% confidence interval (**Equation 11**), which are not obvious because of extremely tight confidence intervals. Numbers on the right corner of each plot are correlation coefficients between mean genetic gains estimated based on derivation and simulation.

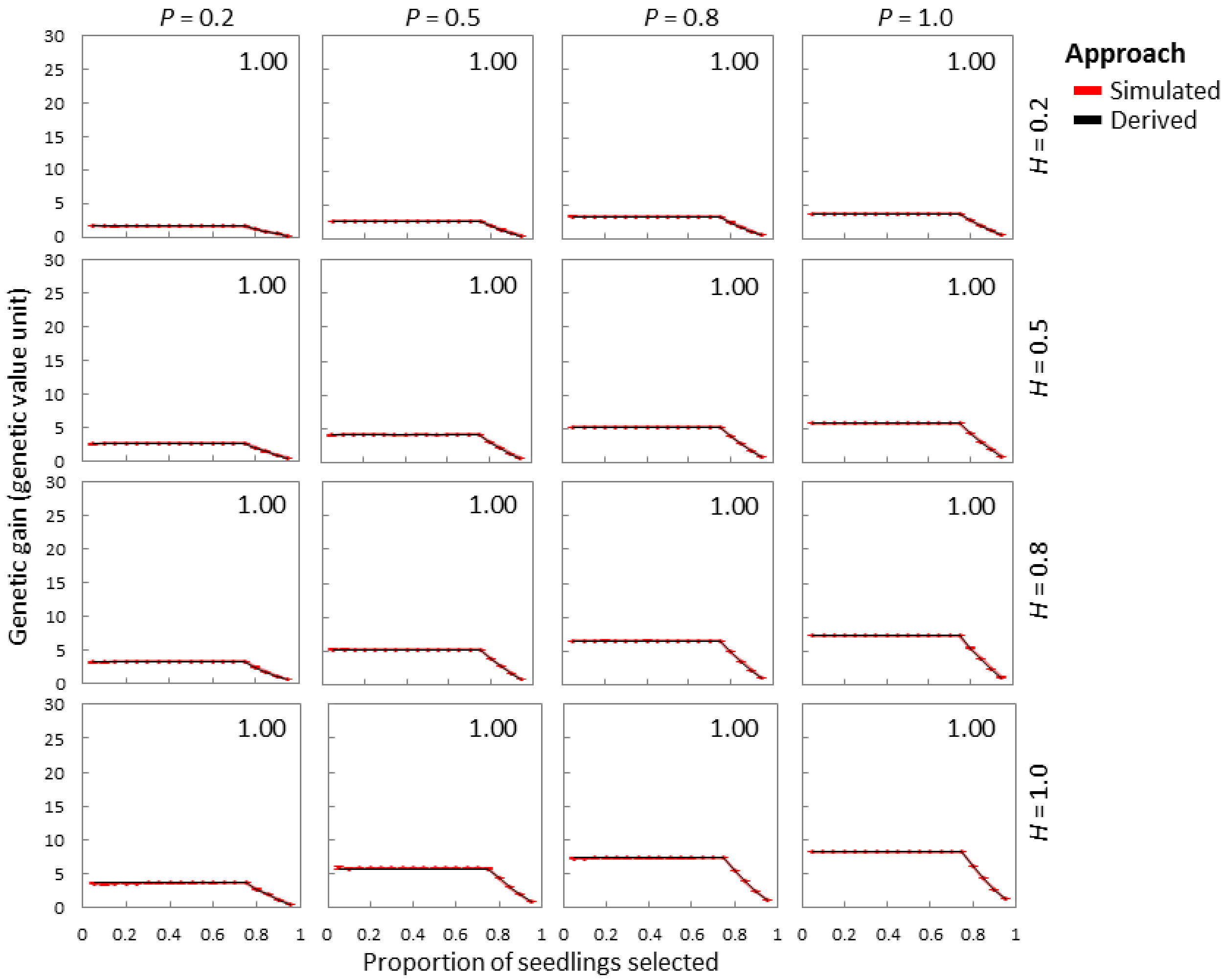

Fig. S3 (c) Comparison between derived and simulated optimal genetic gains from *marker -only* seedling selection for the population with three segregating genotypes with complete dominance ($d_3 = a_3$). Each plot represents a selection scenario with a given broad-sense heritability (*H*) of the trait and predictiveness (*P*) of the DNA test. In each plot, the X axis indicates the proportion of seedlings selected in the end of seedling selection, ranging from 0.05 to 0.95. The Y axis indicates genetic gain from seedling selection based on the unit of simulated genotypic values. Error bars for each data point indicate the 95% confidence interval (**Equation 11**), which are not obvious because of extremely tight confidence intervals. Numbers on the right corner of each plot are correlation coefficients between mean genetic gains estimated based on derivation and simulation.

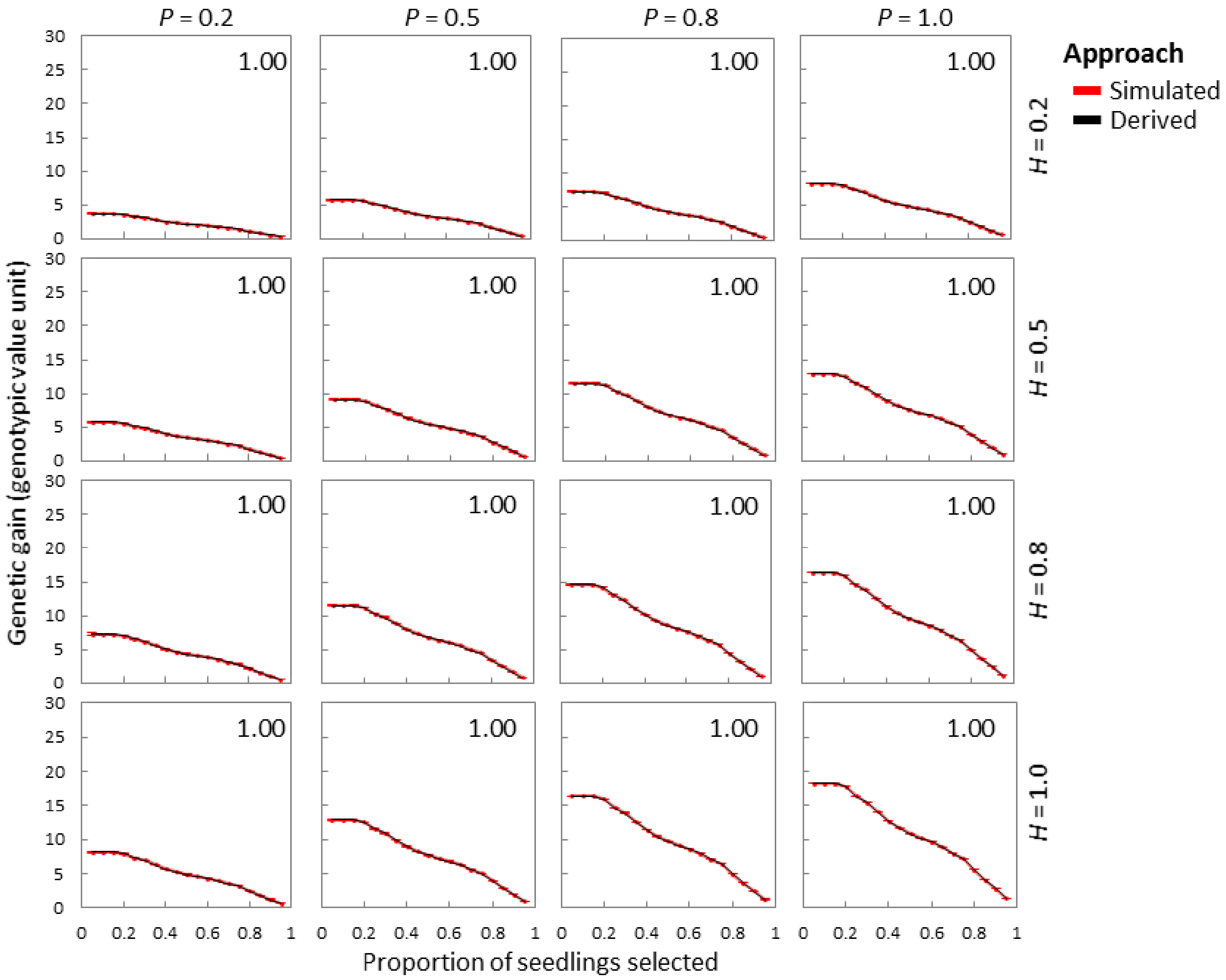

Fig. S3 (d) Comparison between derived and simulated optimal genetic gains from *marker -only* seedling selection for the population with nine segregating genotypes. Each plot represents a selection scenario with a given broad-sense heritability (*H*) of the trait and predictiveness (*P*) of the DNA test. In each plot, the X axis indicates the proportion of seedlings selected in the end of seedling selection, ranging from 0.05 to 0.95. The Y axis indicates genetic gain from seedling selection based on the unit of simulated genotypic values. Error bars for each data point indicate the 95% confidence interval (**Equation 11**), which are not obvious because of extremely tight confidence intervals. Numbers on the right corner of each plot are correlation coefficients between mean genetic gains estimated based on derivation and simulation.

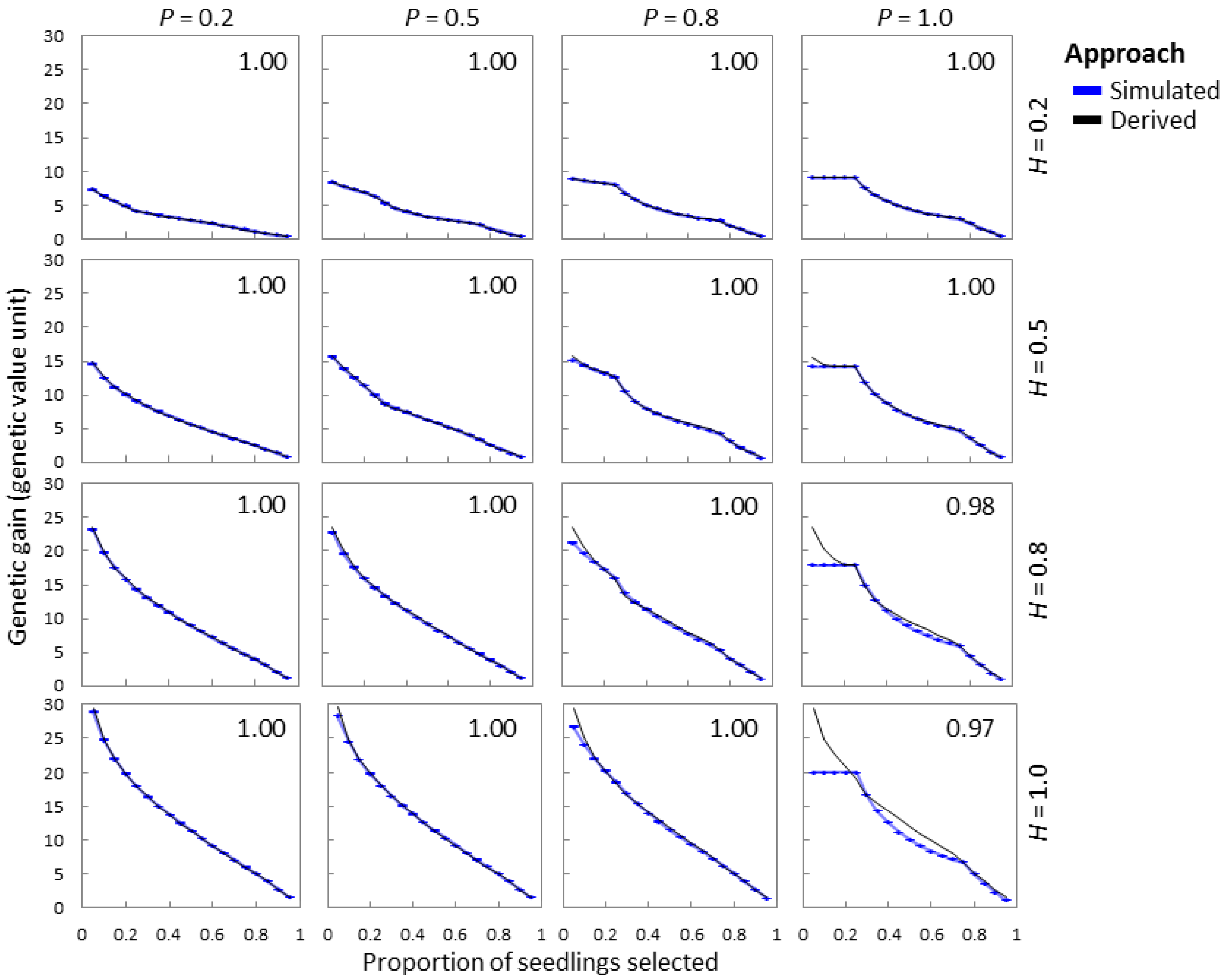

Fig. S4 (a) Comparison between derived and simulated genetic gains from *two-stage* seedling selection for the population with three segregating genotypes without dominance (d_3 = 0). Each plot represents a selection scenario with a given broad-sense heritability (*H*) of the trait and predictiveness (*P*) of the DNA test. In each plot, the X axis indicates the proportion of seedlings selected in the end of seedling selection, ranging from 0.05 to 0.95. The Y axis indicates genetic gain from seedling selection based on the unit of simulated genotypic values. Error bars for each data point indicate the 95% confidence interval (**Equation 11**), which are not obvious because of extremely tight confidence intervals. Numbers on the right corner of each plot are correlation coefficients between mean genetic gains estimated based on derivation and simulation.

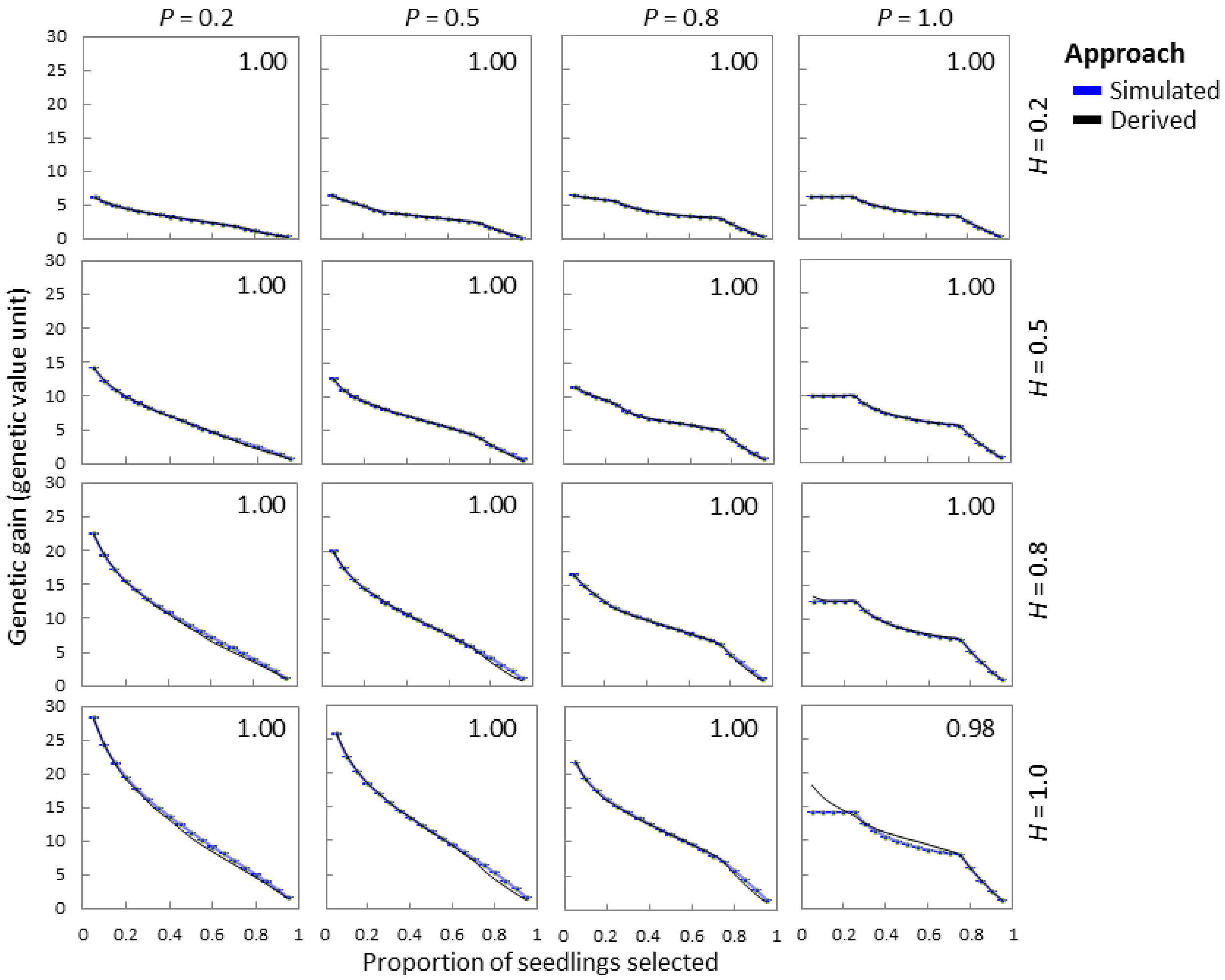

Fig. S4 (b) Comparison between derived and simulated genetic gains from *two-stage* seedling selection for the population with three segregating genotypes with partial dominance ($d_3 = a_3/2$). Each plot represents a selection scenario with a given broad-sense heritability (*H*) of the trait and predictiveness (*P*) of the DNA test. In each plot, the X axis indicates the proportion of seedlings selected in the end of seedling selection, ranging from 0.05 to 0.95. The Y axis indicates genetic gain from seedling selection based on the unit of simulated genotypic values. Error bars for each data point indicate the 95% confidence interval (**Equation 11**), which are not obvious because of extremely tight confidence intervals. Numbers on the right corner of each plot are correlation coefficients between mean genetic gains estimated based on derivation and simulation.

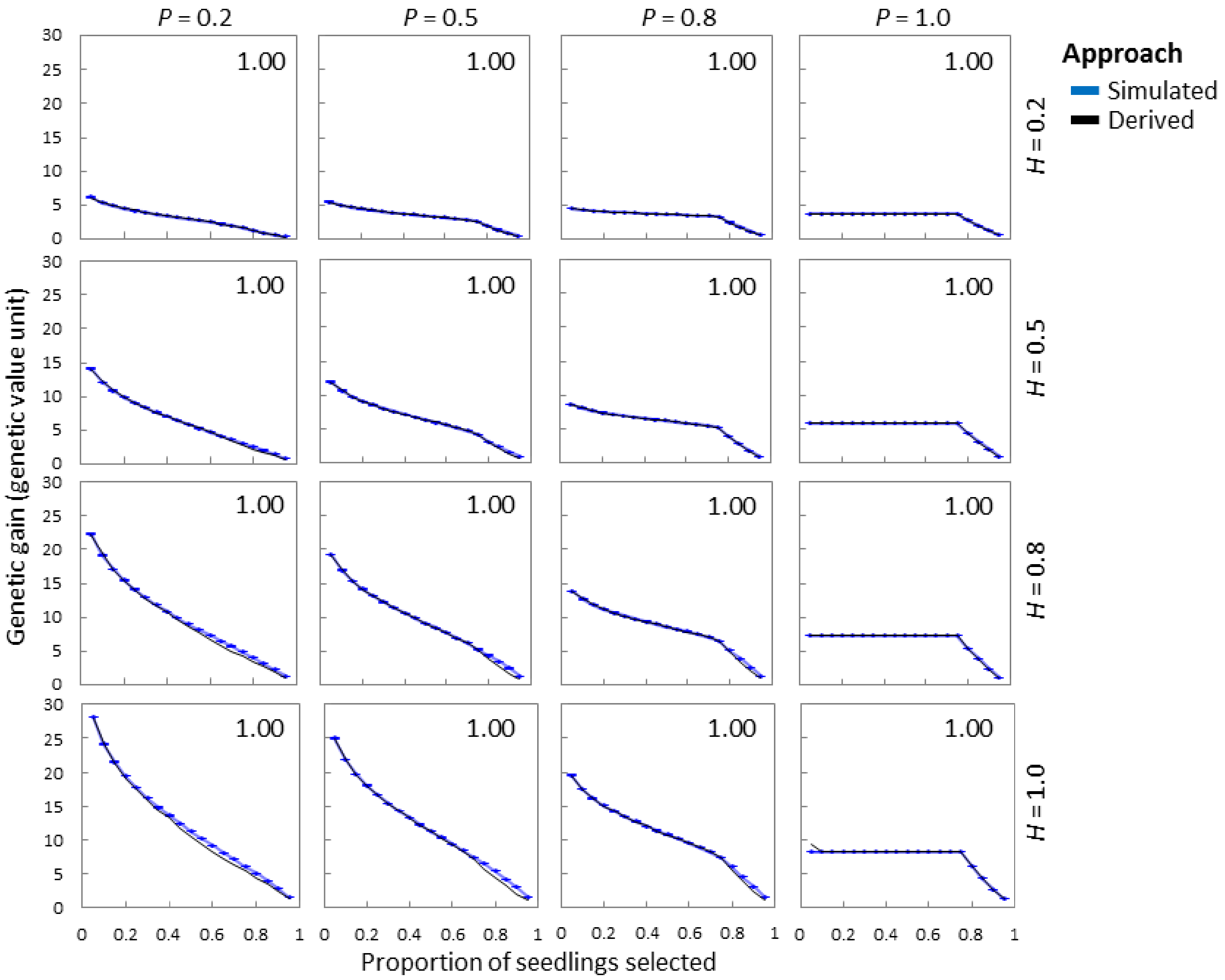

Fig. S4 (c) Comparison between derived and simulated genetic gains from *two-stage* seedling selection for the population with three segregating genotypes with complete dominance ($d_3 = a_3$). Each plot represents a selection scenario with a given broad-sense heritability (*H*) of the trait and predictiveness (*P*) of the DNA test. In each plot, the X axis indicates the proportion of seedlings selected in the end of seedling selection, ranging from 0.05 to 0.95. The Y axis indicates genetic gain from seedling selection based on the unit of simulated genotypic values. Error bars for each data point indicate the 95% confidence interval (**Equation 11**), which are not obvious because of extremely tight confidence intervals. Numbers on the right corner of each plot are correlation coefficients between mean genetic gains estimated based on derivation and simulation.

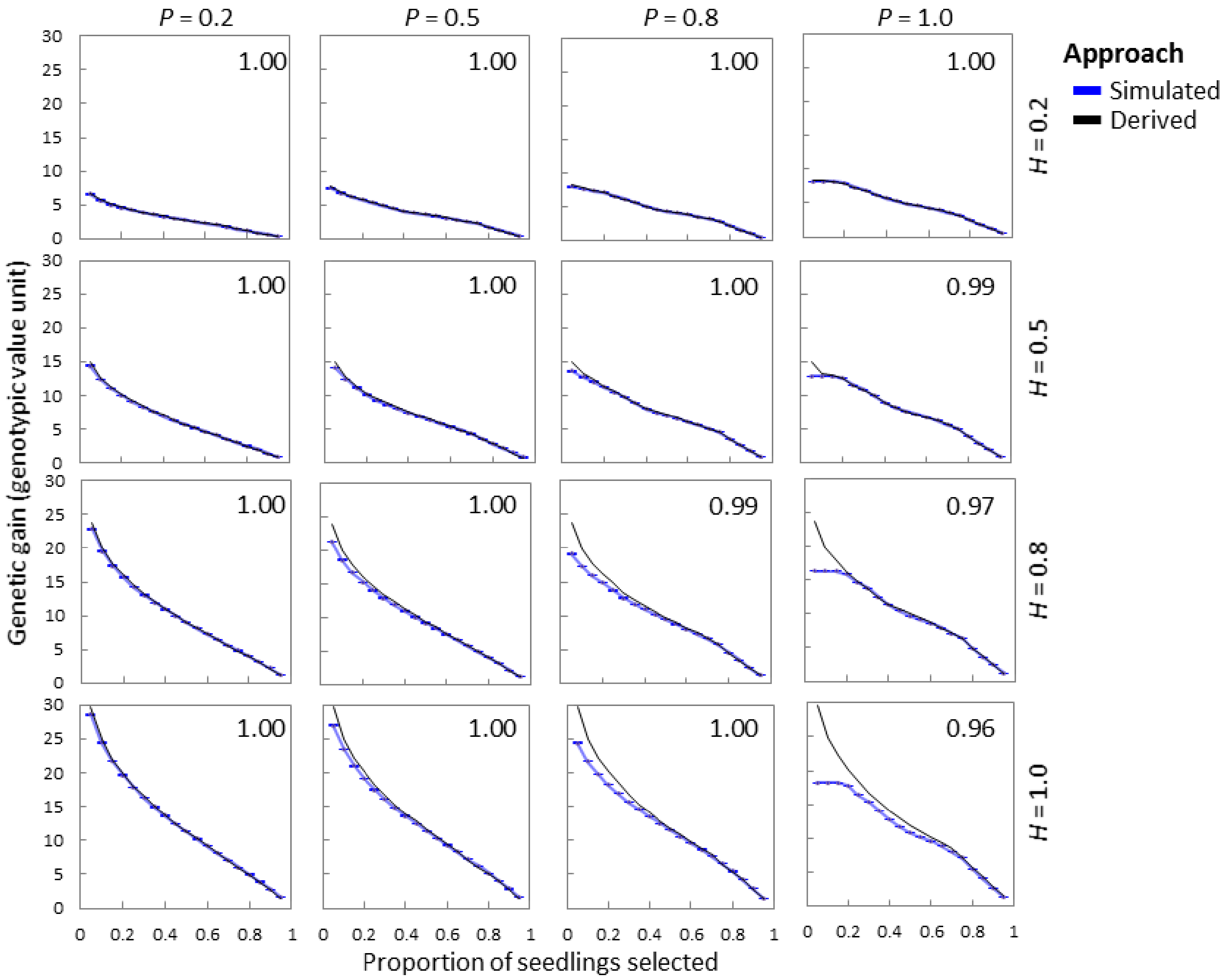

Fig. S4 (d) Comparison between derived and simulated genetic gains from *two-stage* seedling selection for the population with nine segregating genotypes. Each plot represents a selection scenario with a given broad-sense heritability (*H*) of the trait and predictiveness (*P*) of the DNA test. In each plot, the X axis indicates the proportion of seedlings selected in the end of seedling selection, ranging from 0.05 to 0.95. The Y axis indicates genetic gain from seedling selection based on the unit of simulated genotypic values. Error bars for each data point indicate the 95% confidence interval (**Equation 11**), which are not obvious because of extremely tight confidence intervals. Numbers on the right corner of each plot are correlation coefficients between mean genetic gains estimated based on derivation and simulation.

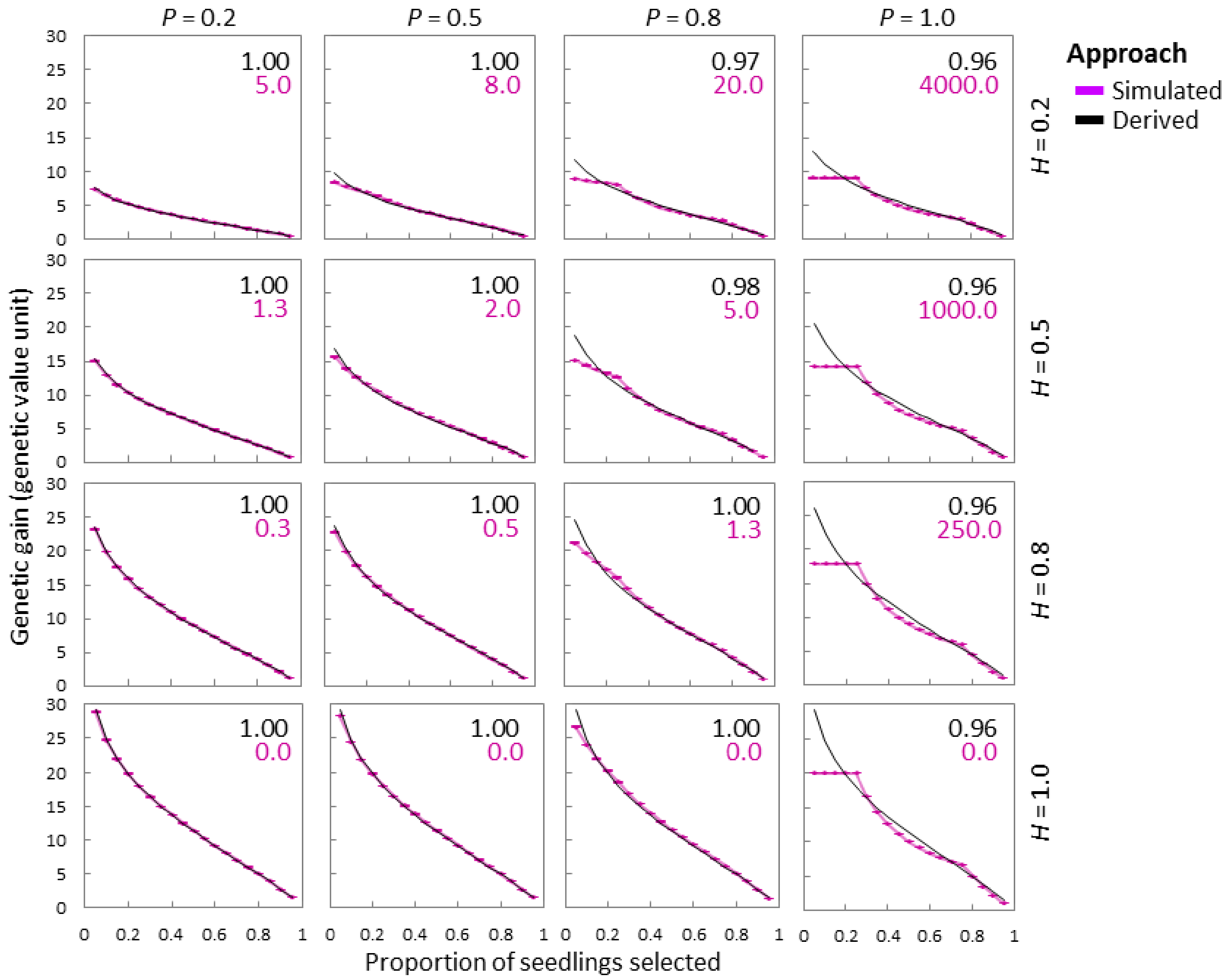

Fig. S5 (a) Comparison between derived and simulated genetic gains from *index* seedling selection for the population with three segregating genotypes without dominance ($d_3 = 0$). Each plot represents a selection scenario with a given broad-sense heritability (*H*) of the trait and predictiveness (*P*) of the DNA test. In each plot, the X axis indicates the proportion of seedlings selected in the end of seedling selection, ranging from 0.05 to 0.95. The Y axis indicates genetic gain from seedling selection based on the unit of simulated genotypic values. Error bars for each data point indicate the 95% confidence interval (**Equation 11**), which are not obvious because of extremely tight confidence intervals. Black numbers on the right corner of each plot are correlation coefficients between mean genetic gains estimated based on derivation and simulation. Pink numbers indicate ratios between weight coefficients of the phenotypic score and marker score in each trait-test scenario.

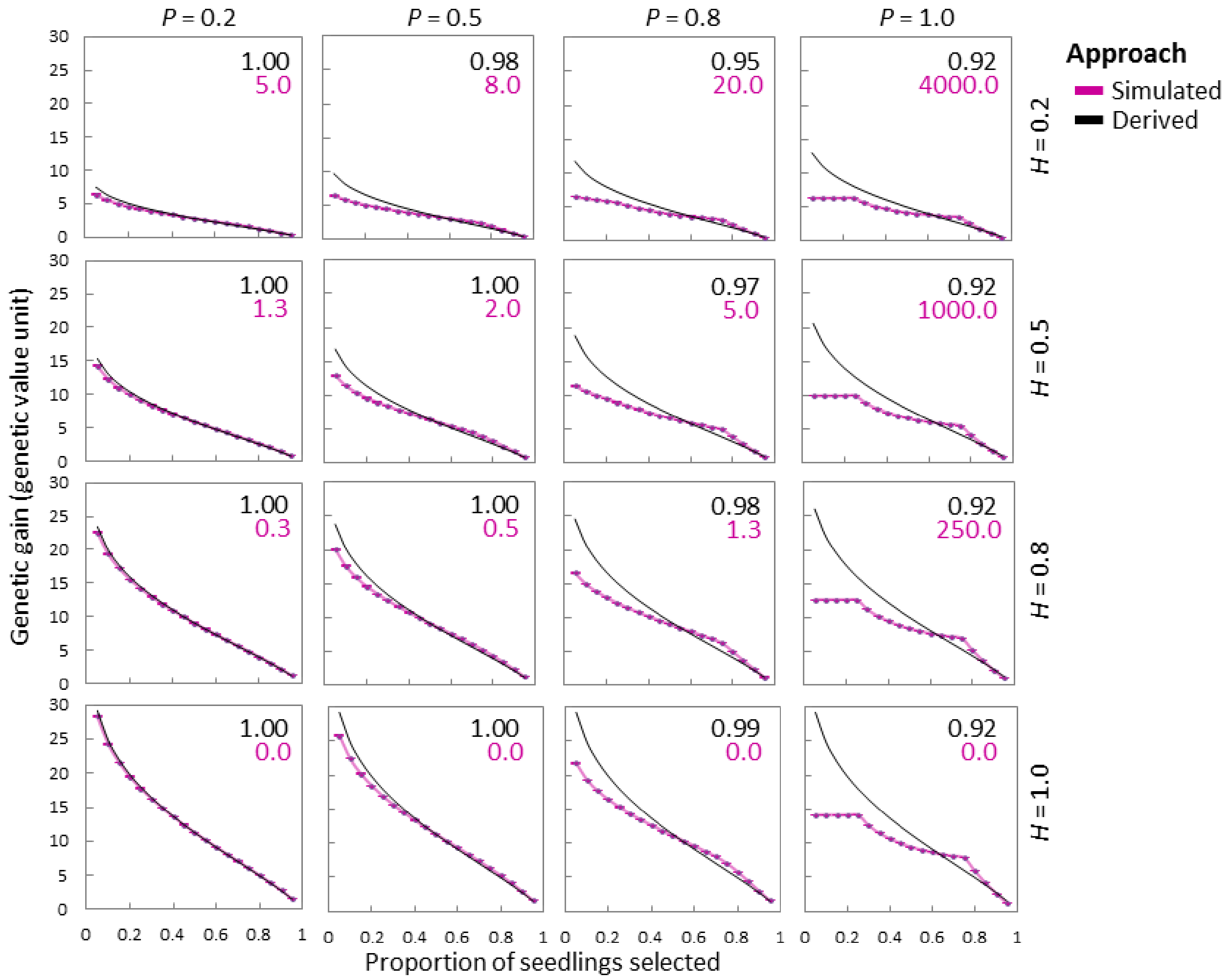

Fig. S5 (b) Comparison between derived and simulated genetic gains from *index* seedling selection for the population with three segregating genotypes with partial dominance ($d_3 = a_3/2$). Each plot represents a selection scenario with a given broad-sense heritability (*H*) of the trait and predictiveness (*P*) of the DNA test. In each plot, the X axis indicates the proportion of seedlings selected in the end of seedling selection, ranging from 0.05 to 0.95. The Y axis indicates genetic gain from seedling selection based on the unit of simulated genotypic values. Error bars for each data point indicate the 95% confidence interval (**Equation 11**), which are not obvious because of extremely tight confidence intervals. Black numbers on the right corner of each plot are correlation coefficients between mean genetic gains estimated based on derivation and simulation. Pink numbers indicate ratios between weight coefficients of the phenotypic score and marker score in each trait-test scenario.

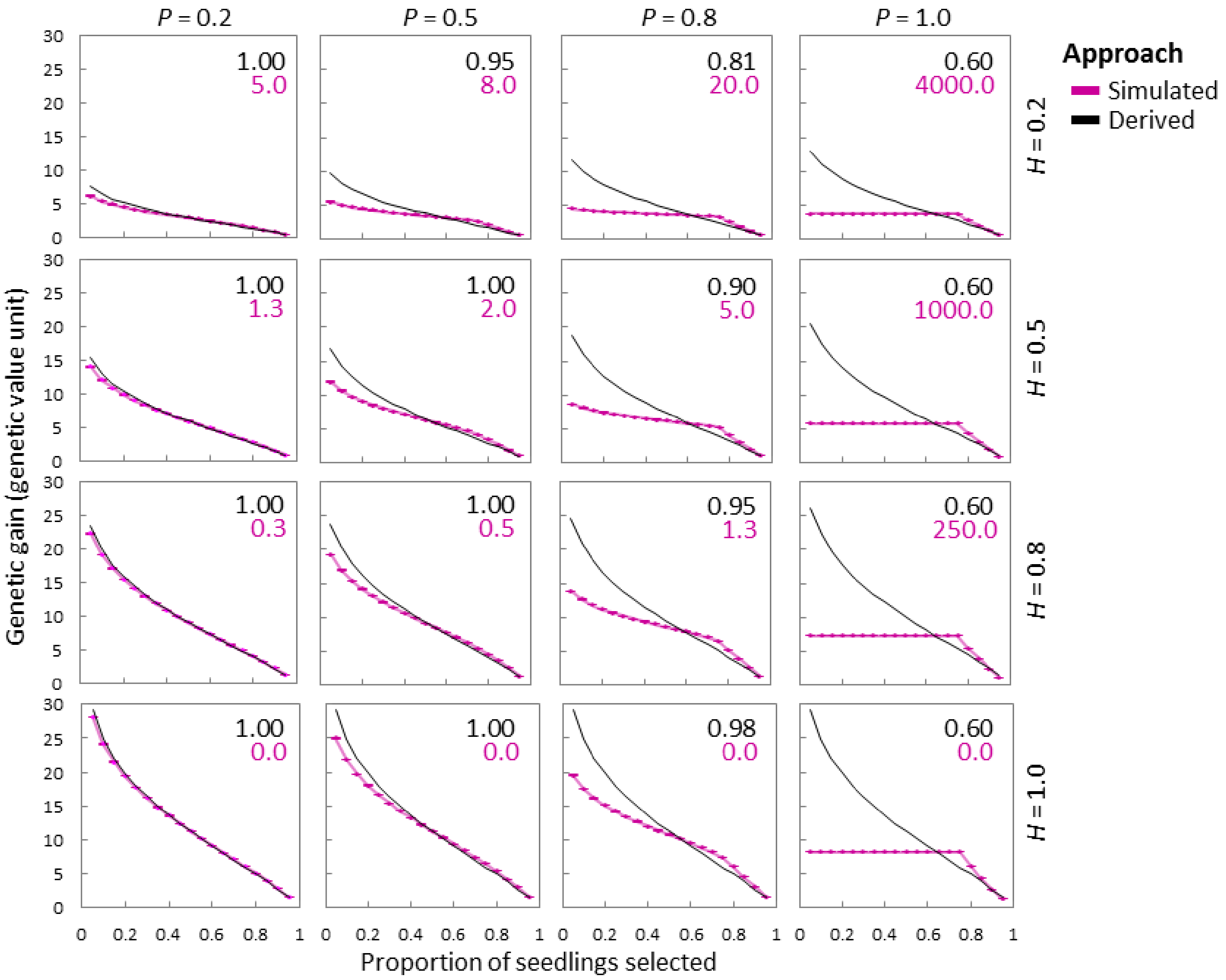

Fig. S5 (c) Comparison between derived and simulated genetic gains from *index* seedling selection for the population with three segregating genotypes with complete dominance ($d_3 = a_3$). Each plot represents a selection scenario with a given broad-sense heritability (*H*) of the trait and predictiveness (*P*) of the DNA test. In each plot, the X axis indicates the proportion of seedlings selected in the end of seedling selection, ranging from 0.05 to 0.95. The Y axis indicates genetic gain from seedling selection based on the unit of simulated genotypic values. Error bars for each data point indicate the 95% confidence interval (**Equation 11**), which are not obvious because of extremely tight confidence intervals. Black numbers on the right corner of each plot are correlation coefficients between mean genetic gains estimated based on derivation and simulation. Pink numbers indicate ratios between weight coefficients of the phenotypic score and marker score in each trait-test scenario.

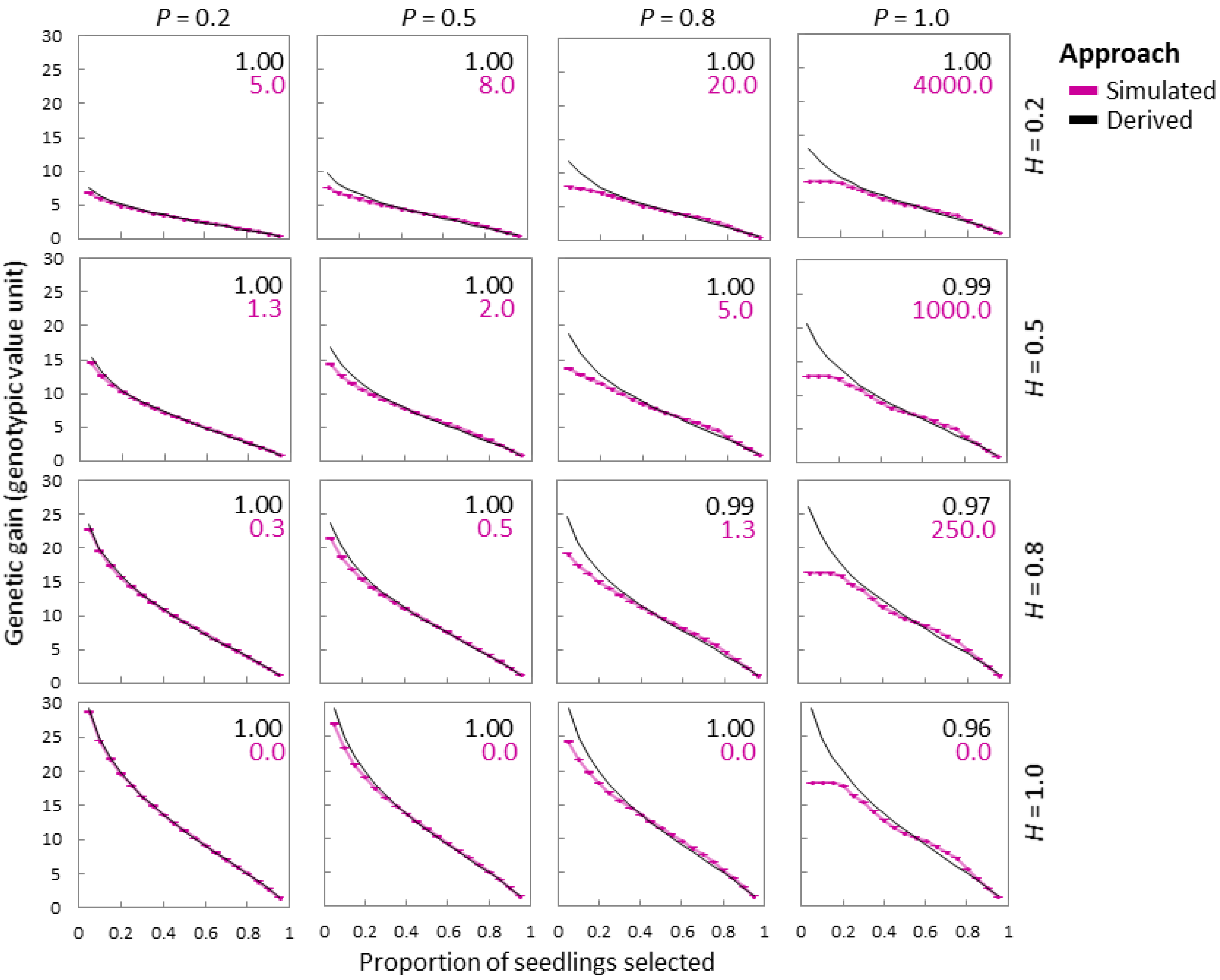

Fig. S5 (d) Comparison between derived and simulated genetic gains from *index* seedling selection for the population with nine segregating genotypes. Each plot represents a selection scenario with a given broad-sense heritability (*H*) of the trait and predictiveness (*P*) of the DNA test. In each plot, the X axis indicates the proportion of seedlings selected in the end of seedling selection, ranging from 0.05 to 0.95. The Y axis indicates genetic gain from seedling selection based on the unit of simulated genotypic values. Error bars for each data point indicate the 95% confidence interval (**Equation 11**), which are not obvious because of extremely tight confidence intervals. Black numbers on the right corner of each plot are correlation coefficients between mean genetic gains estimated based on derivation and simulation. Pink numbers indicate ratios between weight coefficients of the phenotypic score and marker score in each trait-test scenario.

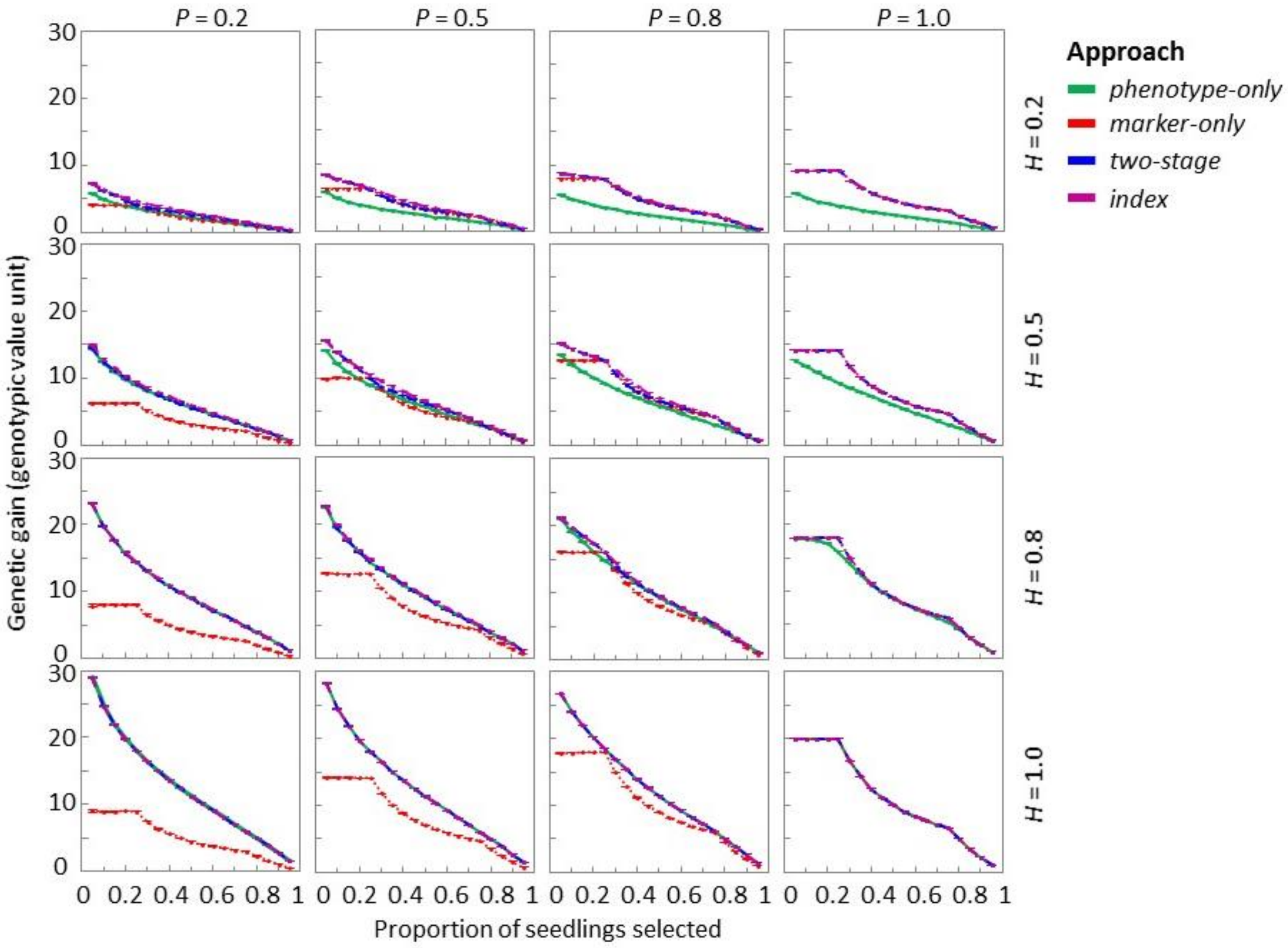

Fig. S6 (a) Simulated genetic gain from alternative seedling selection strategies for the population with three segregating genotypes and no dominance ($d_3 = 0$). Each plot represents a selection scenario with a given broad-sense heritability (H) of the trait and predictiveness (P) of the DNA test. In each plot, the X axis indicates the proportion of seedlings selected in the end of seedling selection, ranging from 0.05 to 0.95. The Y axis indicates genetic gain from seedling selection based on the unit of simulated genotypic values. Error bars for each data point indicate the 95% confidence interval (**Equation 11**), which are not obvious because of extremely tight confidence intervals.

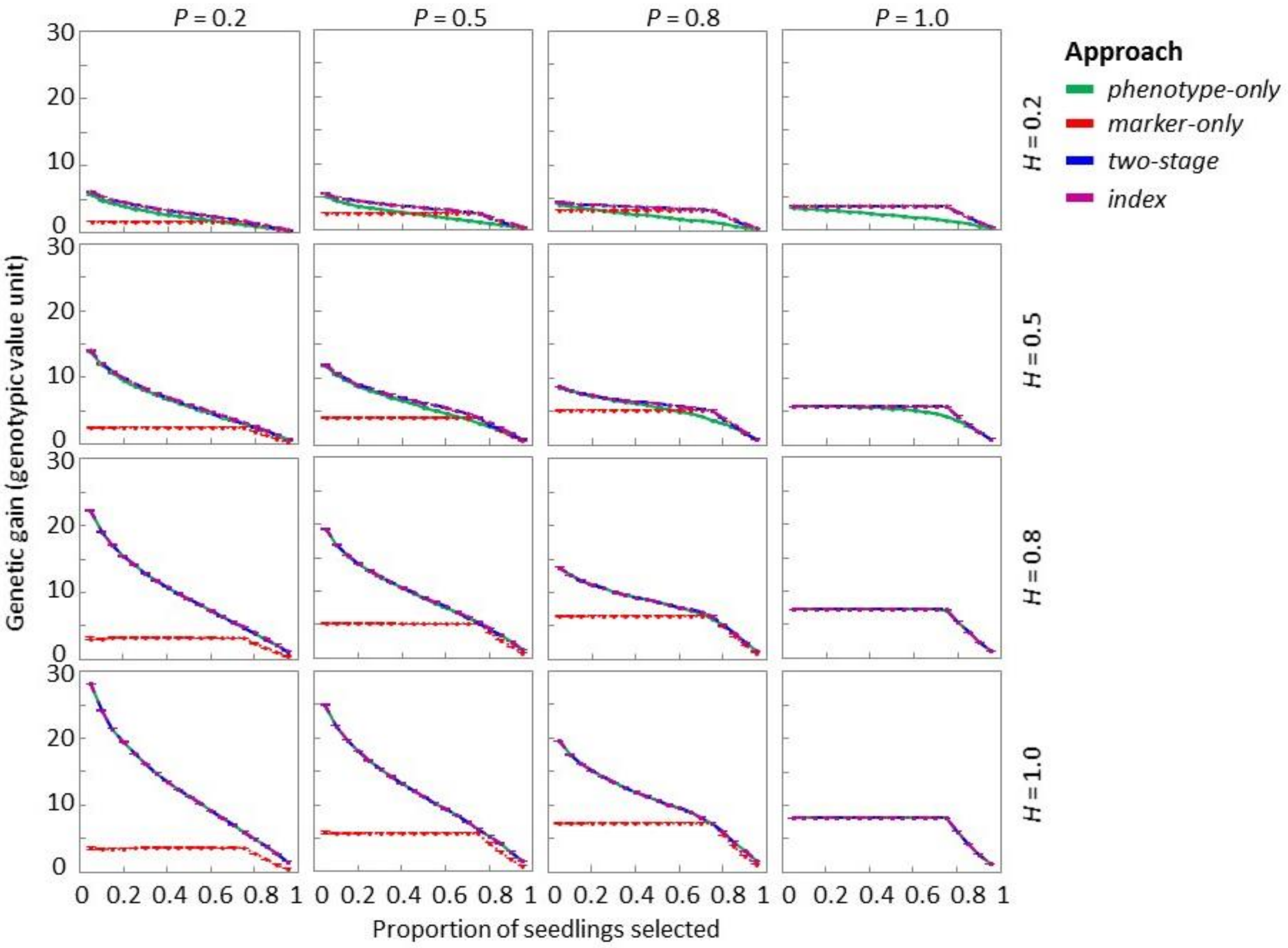

Fig. S6 (b) Simulated genetic gain from alternative seedling selection strategies for the population with three segregating genotypes and complete dominance ($d_3 = a_3$). Each plot represents a selection scenario with a given broad-sense heritability (H) of the trait and predictiveness (P) of the DNA test. In each plot, the X axis indicates the proportion of seedlings selected in the end of seedling selection, ranging from 0.05 to 0.95. The Y axis indicates genetic gain from seedling selection based on the unit of simulated genotypic values. Error bars for each data point indicate the 95% confidence interval (**Equation 11**), which are not obvious because of extremely tight confidence intervals.

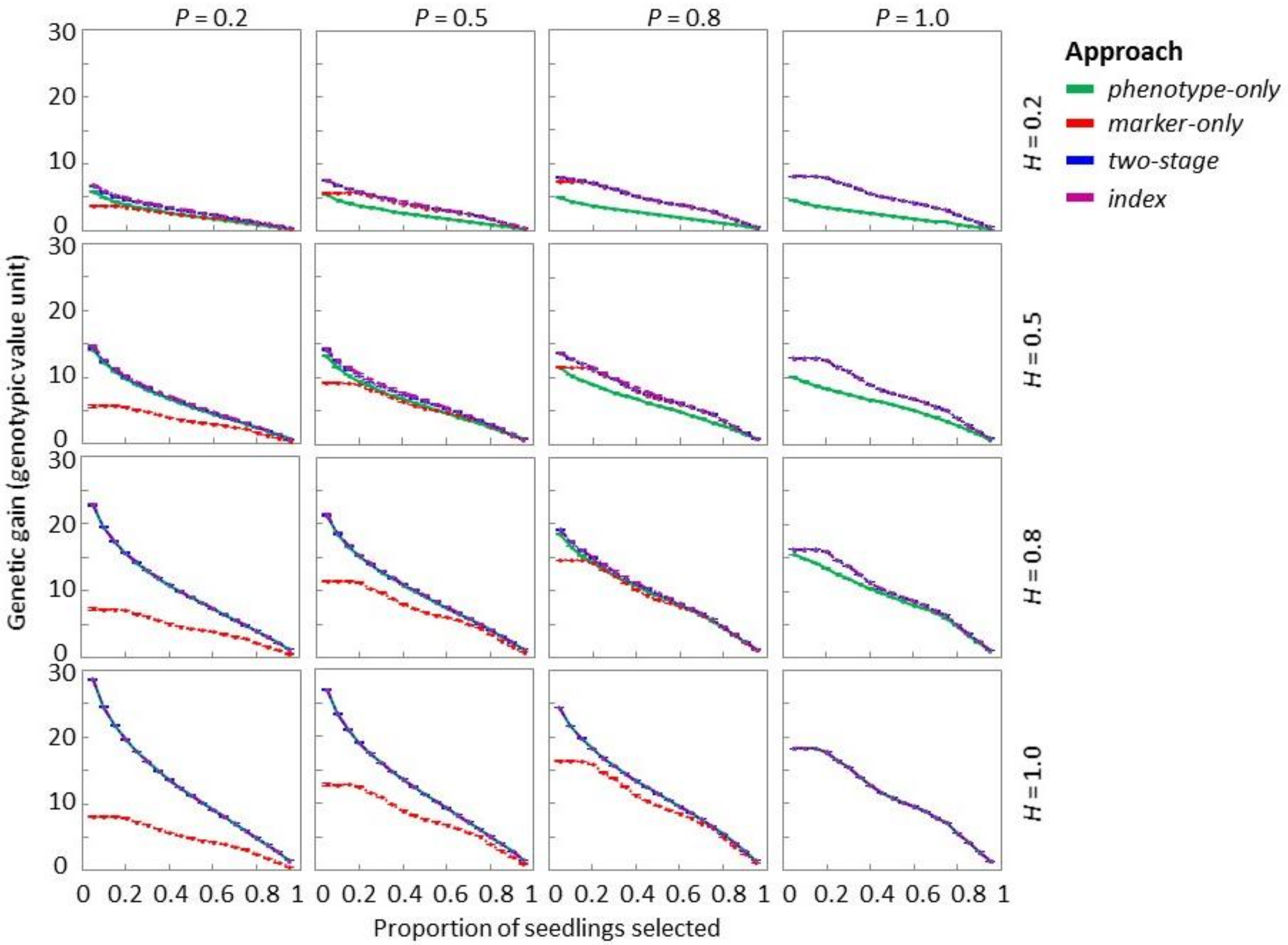

Fig. S6 (c) Simulated genetic gain from alternative seedling selection strategies for the population with nine segregating genotypes. Each plot represents a selection scenario with a given broad-sense heritability (*H*) of the trait and predictiveness (*P*) of the DNA test. In each plot, the X axis indicates the proportion of seedlings selected in the end of seedling selection, ranging from 0.05 to 0.95. The Y axis indicates genetic gain from seedling selection based on the unit of simulated genotypic values. Error bars for each data point indicate the 95% confidence interval (**Equation 11**), which are not obvious because of extremely tight confidence intervals.

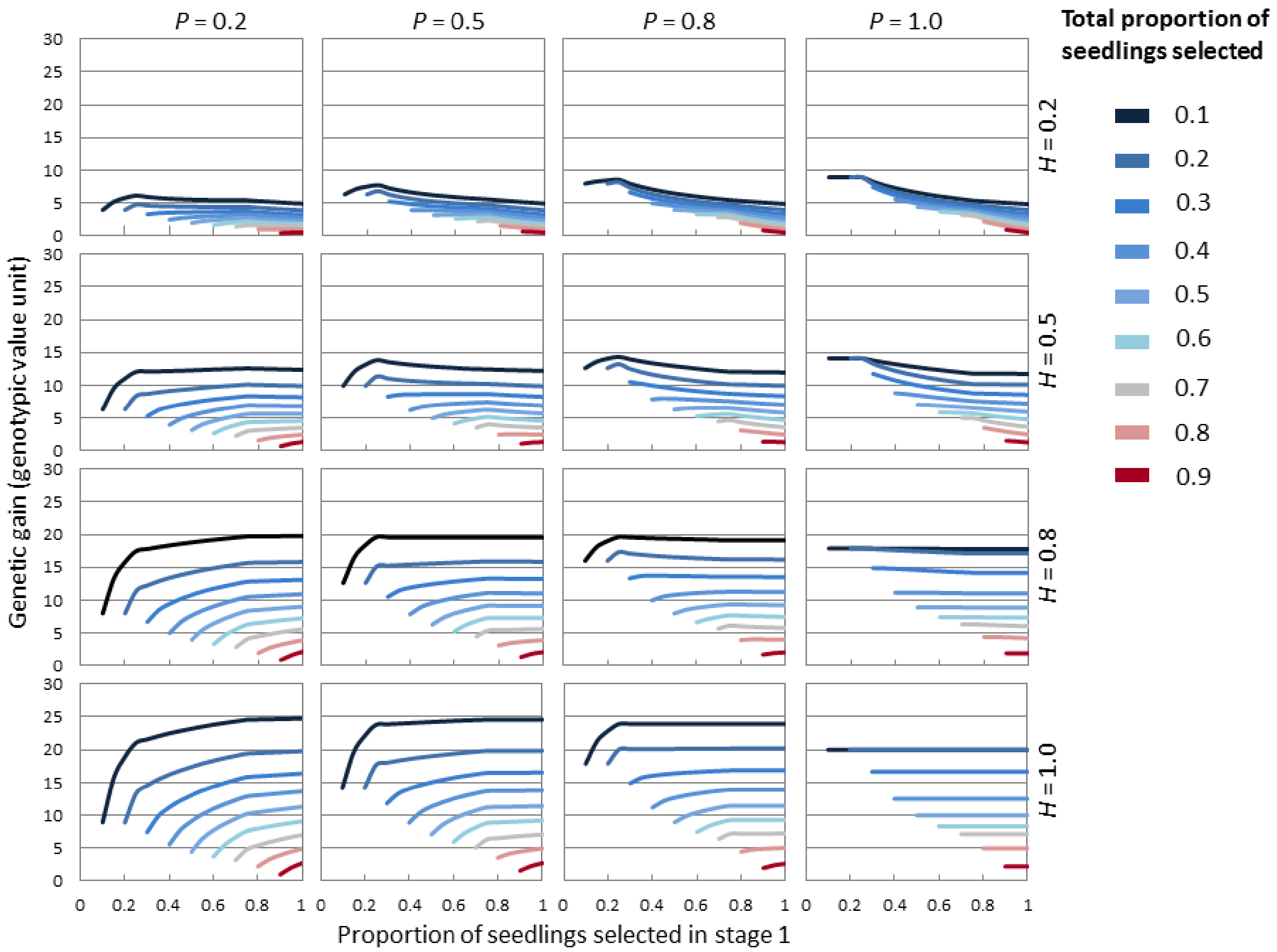

Fig. S7 (a) Simulated genetic gain from *two-stage* seedling selection for the population with three segregating genotypes and no dominance ($d_3 = 0$). Each plot represents a selection scenario with a given broad-sense heritability (H) of the trait and predictiveness (P) of the DNA test. In each plot, the X axis indicates the proportion of seedlings selected in the first stage, and the Y axis indicates simulated genetic gain from *two-stage* seedling selection based on SP_M.

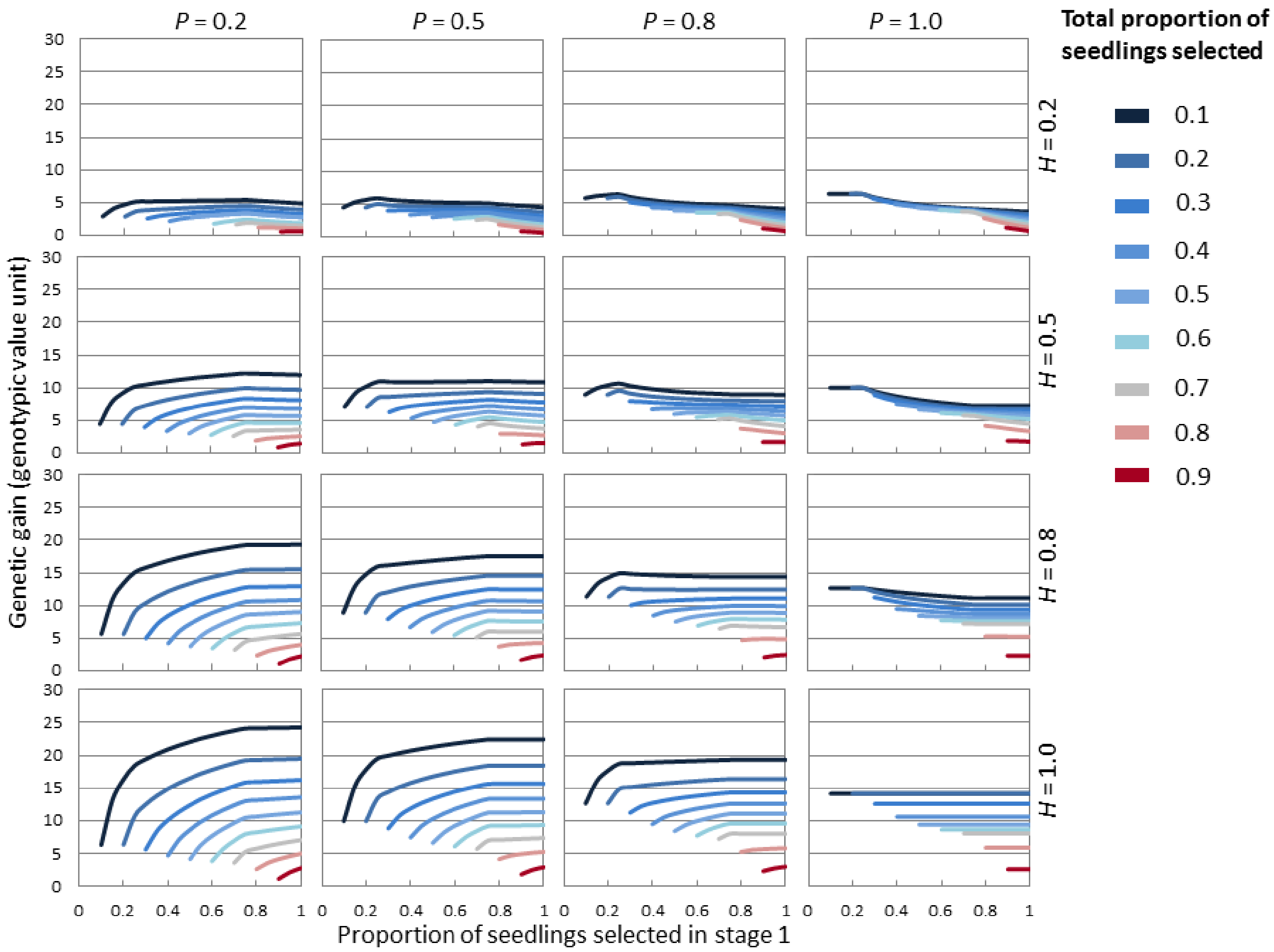

Fig. S7 (b) Simulated genetic gain from *two-stage* seedling selection for the population with three segregating genotypes and complete dominance ($d_3 = a_3$). Each plot represents a selection scenario with a given broad-sense heritability (*H*) of the trait and predictiveness (*P*) of the DNA test. In each plot, the X axis indicates the proportion of seedlings selected in the first stage, and the Y axis indicates simulated genetic gain from *two-stage* seedling selection based on SP_M.

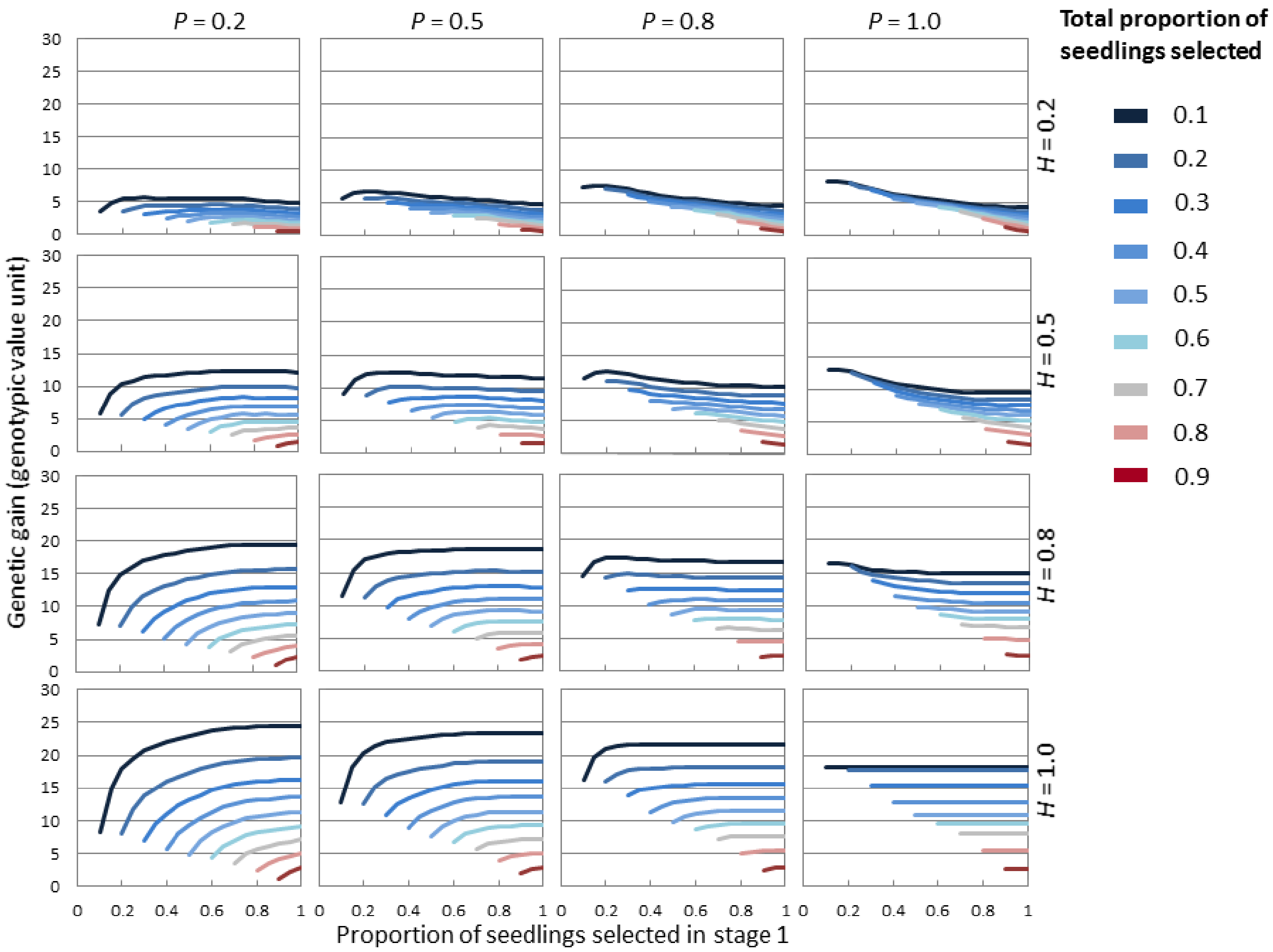

Fig. S7 (c) Simulated genetic gain from *two-stage* seedling selection for the population with nine segregating genotypes. Each plot represents a selection scenario with a given broad-sense heritability (*H*) of the trait and predictiveness (*P*) of the DNA test. In each plot, the X axis indicates the proportion of seedlings selected in the first stage, and the Y axis indicates simulated genetic gain from *two-stage* seedling selection based on SP_M.

Simulation source code for the population with three segregating genotypes and partial dominance

```
###############function datageration#####################
# input: H, RGG, info; return: ori <- VG, VGQ, VGB, VE, sb, se, a, addq, gq, gsNo
# info include:
# info$gNo: number of genotype
# info$p1: proportion of genotype1
# info$p2: proportion of genotype2
# info$p3: proportion of genotype3
# info$seedno: seed number for rand()
# info$tNo: total seedling number
# info$midPoint: mid-point value beweetn genotypic value of QQ and qq
# info$VP: phenotypic variance
# info$dIndex: index of dominant effect, d = info$dIndex * a
datageneration <- function(H, RGG, info){

  # creat a list of genotype distribution c(g1, g2, g3)
  for (i in 1:info$gNo){
    if (i == 1){
      gpercent <-info[1, i + 1]
    }else{
      gpercent <- c(gpercent, info[1, i + 1] )
    }
  }

  # calculate variance
  VG <- info$VP * H
  VGQ <- VG * RGG
  VGB <- VG - VGQ
  VE <- info$VP - VG

  sb <- VGB^.5
  se <- VE^.5

  # calculate selection index, use 0.999 when RGG == 1
  bz <- 1
  if (RGG == 1)
  {
    bm <- (1/H - 1)/ (1 - 0.999)  # if RGG == 1, use 0.999 to calculate bm
  }else
  {
    bm <- (1/H - 1)/(1 - RGG)
  }
```

```
  ###########different for different cases################

  a <- (4*VGQ/(2 + (info$dIndex)^2))^0.5
  addq <-c(-a, a * info$dIndex, a) # genotypic effect of qq, Qq, QQ after substracting midpoint value
  mean <- info$midPoint + info$dIndex * a / 2 # population mean

  ##########################################################
  # calculate seedling number for each genotype
  SeedNo <- 0 # No. of seedlings in each genotype
  sequenceNo <- 0 # culmulative No. of seedlings in the first n genotypes
  count <- 0
  gqq <- 0

  for (i in 1:info$gNo){
    SeedNo[i] = info$tNo * gpercent[i]
    count <- count + SeedNo[i]
    sequenceNo[i] <- count # starting/ending No. of genotype i: sequenceNo[i-1] + 1, sequenceNo[i]
  }

  # generate QLT genetic values
  gq <- rep(addq[1], SeedNo[1]) # SeedNo[i] is the number of seedlings with ith genotype
  gsNo <- rep(1, SeedNo[1]) # the sequence No. of the genotype



  for (i in 2:info$gNo){
    gq <- c(gq, rep(addq[i], SeedNo[i]))
    gsNo <- c(gsNo, rep(i, SeedNo[i]))

  }

  ori_scen <- list('VG' = VG, 'VGQ' = VGQ, 'VGB' = VGB, 'VE' = VE, 'sb' = sb, 'se' = se, 'a' = a, 'addq' =
addq, 'gq' = gq, 'bz' = bz, 'bm' = bm, 'gsNo'= gsNo)
  scen_sum <- list('VG' = VG, 'VGQ' = VGQ, 'VGB' = VGB, 'VE' = VE, 'sb' = sb, 'se' = se, 'a' = a, 'd' = a *
info$dIndex)
  write.csv(scen_sum, file = sprintf("scen_Sum_H%.2fP%.2f.csv", H, RGG), row.names = F)
  return(ori_scen)
}

########################################function sort() to generate g,p values and
sort###################
sort <- function(info, ori_scen){
  # generate genetic values
  gb <- c(rnorm(info$tNo, mean = 0, sd = ori_scen$sb))
  g <- rep(info$midPoint, info$tNo) + ori_scen$gq + gb
```

```
  # generate phenotypic distribution
  e <- c(rnorm(info$tNo, mean = 0, sd = ori_scen$se))
  p <- g + e

  # generate index
  i <- ori_scen$bz*p + ori_scen$bm*ori_scen$gq

  # creat data frame of gq, g, p, gsNo
  oridataframe <- data.frame(ori_scen$gq, g, p, i, ori_scen$gsNo)

  # sort ori according to different standards
  # sort data frame according to g_value, decreasing
  ori_gq_sort <- oridataframe[order(-ori_scen$gq),]
  # sort data frame according to g_value, decreasing
  ori_g_sort <- oridataframe[order(-g),]
  # sort data frame according to p_value, decreasing
  ori_p_sort <- oridataframe[order(-p),]
  # sort data frame according to index, decreasing
  ori_i_sort <- oridataframe[order(-i),]

  ori_data <- list('gb'= gb, 'g' = g,'e' = e, 'p' = p, 'i' = i,'oridataframe' = oridataframe,
                   'gq_sort'= ori_gq_sort, 'p_sort'= ori_p_sort, 'i_sort' = ori_i_sort)

  #write.csv(oridataframe, file = sprintf("Oridata_H%.2fP%.2f.csv", H, RGG), row.names = F)
  return(ori_data)
}

###############################function selection###################
# simulate g, p and conduct selection

selection <- function(info, ori, ori_data, Tsr){
  #################different for different No. of genotypes #############
  Msr1_seq <- seq(0.05, 1, 0.05) # Msr1 ranges from 0.05 to 1
  dgMASSlist <- rep(NA, 20)
  #########################################################################

  # summarize the population
  mp <- sapply(ori_data$oridataframe[3], mean) # mean phenotypic value of the whole population
  mg <- sapply(ori_data$oridataframe[2], mean) # mean genotypic value of the whole population

  # select using TSS and one-stage MASS
  # TSS, select for the top "Tsr" percentage based on phenotype
  TSS <- ori_data$p_sort[c(seq(1, Tsr*length(ori_data$p), 1)),]
```

```
  # summarize TSS
  mgTSS <- sapply(TSS[2], mean) # mean genotypic value of selected ones in TSS
  # calculate gain in TSS
  dgTSS <- mgTSS - mg # genetic gain in TSS

  # index selection
  Index <- ori_data$i_sort[c(seq(1, Tsr*length(ori_data$p), 1)),]
  # mean genotypic value of selected ones in index
  mgIndex <- sapply(Index[2], mean)
  # calculate gain in TSS
  dgIndex <- mgIndex - mg # genetic gain in TSS

  # One-stage MASS, select for the top "Tsr" percentage only based on marker genotype
  DNA <- ori_data$gq_sort[c(seq(1, Tsr*length(ori_data$p), 1)),]
  # summarize one-stage MASS
  mgDNA <- sapply(DNA[2], mean) # mean genotypic value of selected ones in one-stage MASS
  # calculate gain in one-stage MASS
  dgDNA <- mgDNA - mg # genetic gain in one-stage MASS

  # two-stage selection
  for (Msr1 in Msr1_seq){
    if(Msr1 < Tsr){
      if(Msr1 == 0.05){
        dgMASSlist <- NA
      }else{
        dgMASSlist <- c(dgMASSlist, NA)
      }
    }else{
      Msr2 <- Tsr/Msr1

      # First stage of MASS, select for the top "Msr1" percentage, based on marker genotype
      MASS1 <- ori_data$gq_sort[c(seq(1, Msr1*length(ori_data$p), 1)),]
      # Sort individuals after MASS1 based on phenotype
      MASS1_p_sort <- MASS1[order(-MASS1$p),]
      # Second stage of MASS, select for the top "Msr2" percentage based on phenotype
      MASS2 <- MASS1_p_sort[c(seq(1, Msr2*length(MASS1_p_sort$p), 1)),]

      #summarize two-stage MASS
      mgMASS <- sapply(MASS2[2], mean) # mean phenotypic value of selected ones in the first stage of
MASS
      dgMASS <- mgMASS - mg

      if(Msr1 == 0.05){
        dgMASSlist <- dgMASS
```

```
      }else{
        dgMASSlist <- c(dgMASSlist, dgMASS)
      }
    }

  } # end of for (Msr1 in Msr1_seq)


  sel1 <- list('dgTSS' = dgTSS, 'dgDNA' = dgDNA, 'dgIndex' = dgIndex, 'dgMASS' = dgMASSlist)
  return(sel1)
}

#################################main######################
filename <- 'Input-HR-d-a.csv'
# get input from csv file: seedno, tNo, mean, gNo, VP, p1, p2, p3, sratio
info <- read.csv(file = filename, head = TRUE, sep = ',')
set.seed(info$seedno)

#H_seq <- c(0.2)
#R_seq <- c(0.2)
H_seq <- c(0.2,0.5,0.8,1)
R_seq <- c(0.2,0.5,0.8,1)

repno <- 1000 # number of replications, should be 1000
sceno <- length(H_seq) *length(R_seq) # the total number of scenarios
scecount <- 0 # count the number of scenarios

######################### different for different cases##################
Tsr_seq <- seq(0.05, 0.95, 0.05) # selection threshold ranges from 0.05 to 0.95
NoTsr <- 19 # No. of different Tsr=((0.95-0.05)/0.05+1)
##########################################################################

#defining matrices for mdgTSS, mdgDNA, mdgMASS
Hmat <- matrix(rep(NA, NoTsr*sceno), NoTsr*sceno, 1) # a matrix of H
Rmat <- matrix(rep(NA, NoTsr*sceno), NoTsr*sceno, 1) # a matrix of R
Tsrmat <- matrix(rep(NA, NoTsr*sceno), NoTsr*sceno, 1) # a matrix of Tsr

mTSS <- matrix(rep(NA, NoTsr*sceno), NoTsr*sceno, 1)
mDNA <- matrix(rep(NA, NoTsr*sceno), NoTsr*sceno, 1)
mIndex <- matrix(rep(NA, NoTsr*sceno), NoTsr*sceno, 1)
mMASS0.05 <- matrix(rep(NA, NoTsr*sceno), NoTsr*sceno, 1)
mMASS0.1 <- matrix(rep(NA, NoTsr*sceno), NoTsr*sceno, 1)
mMASS0.15 <- matrix(rep(NA, NoTsr*sceno), NoTsr*sceno, 1)
mMASS0.2 <- matrix(rep(NA, NoTsr*sceno), NoTsr*sceno, 1)
```

```
mMASS0.25 <- matrix(rep(NA, NoTsr*sceno), NoTsr*sceno, 1)
mMASS0.3 <- matrix(rep(NA, NoTsr*sceno), NoTsr*sceno, 1)
mMASS0.35 <- matrix(rep(NA, NoTsr*sceno), NoTsr*sceno, 1)
mMASS0.4 <- matrix(rep(NA, NoTsr*sceno), NoTsr*sceno, 1)
mMASS0.45 <- matrix(rep(NA, NoTsr*sceno), NoTsr*sceno, 1)
mMASS0.5 <- matrix(rep(NA, NoTsr*sceno), NoTsr*sceno, 1)
mMASS0.55 <- matrix(rep(NA, NoTsr*sceno), NoTsr*sceno, 1)
mMASS0.6 <- matrix(rep(NA, NoTsr*sceno), NoTsr*sceno, 1)
mMASS0.65 <- matrix(rep(NA, NoTsr*sceno), NoTsr*sceno, 1)
mMASS0.7 <- matrix(rep(NA, NoTsr*sceno), NoTsr*sceno, 1)
mMASS0.75 <- matrix(rep(NA, NoTsr*sceno), NoTsr*sceno, 1)
mMASS0.8 <- matrix(rep(NA, NoTsr*sceno), NoTsr*sceno, 1)
mMASS0.85 <- matrix(rep(NA, NoTsr*sceno), NoTsr*sceno, 1)
mMASS0.9 <- matrix(rep(NA, NoTsr*sceno), NoTsr*sceno, 1)
mMASS0.95 <- matrix(rep(NA, NoTsr*sceno), NoTsr*sceno, 1)
mMASS1.0 <- matrix(rep(NA, NoTsr*sceno), NoTsr*sceno, 1)

varTSS <- matrix(rep(NA, NoTsr*sceno), NoTsr*sceno, 1)
varDNA <- matrix(rep(NA, NoTsr*sceno), NoTsr*sceno, 1)
varIndex <- matrix(rep(NA, NoTsr*sceno), NoTsr*sceno, 1)
varMASS0.05 <- matrix(rep(NA, NoTsr*sceno), NoTsr*sceno, 1)
varMASS0.1 <- matrix(rep(NA, NoTsr*sceno), NoTsr*sceno, 1)
varMASS0.15 <- matrix(rep(NA, NoTsr*sceno), NoTsr*sceno, 1)
varMASS0.2 <- matrix(rep(NA, NoTsr*sceno), NoTsr*sceno, 1)
varMASS0.25 <- matrix(rep(NA, NoTsr*sceno), NoTsr*sceno, 1)
varMASS0.3 <- matrix(rep(NA, NoTsr*sceno), NoTsr*sceno, 1)
varMASS0.35 <- matrix(rep(NA, NoTsr*sceno), NoTsr*sceno, 1)
varMASS0.4 <- matrix(rep(NA, NoTsr*sceno), NoTsr*sceno, 1)
varMASS0.45 <- matrix(rep(NA, NoTsr*sceno), NoTsr*sceno, 1)
varMASS0.5 <- matrix(rep(NA, NoTsr*sceno), NoTsr*sceno, 1)
varMASS0.55 <- matrix(rep(NA, NoTsr*sceno), NoTsr*sceno, 1)
varMASS0.6 <- matrix(rep(NA, NoTsr*sceno), NoTsr*sceno, 1)
varMASS0.65 <- matrix(rep(NA, NoTsr*sceno), NoTsr*sceno, 1)
varMASS0.7 <- matrix(rep(NA, NoTsr*sceno), NoTsr*sceno, 1)
varMASS0.75 <- matrix(rep(NA, NoTsr*sceno), NoTsr*sceno, 1)
varMASS0.8 <- matrix(rep(NA, NoTsr*sceno), NoTsr*sceno, 1)
varMASS0.85 <- matrix(rep(NA, NoTsr*sceno), NoTsr*sceno, 1)
varMASS0.9 <- matrix(rep(NA, NoTsr*sceno), NoTsr*sceno, 1)
varMASS0.95 <- matrix(rep(NA, NoTsr*sceno), NoTsr*sceno, 1)
varMASS1.0 <- matrix(rep(NA, NoTsr*sceno), NoTsr*sceno, 1)

clTSS <- matrix(rep(NA, NoTsr*sceno), NoTsr*sceno, 1)
clDNA <- matrix(rep(NA, NoTsr*sceno), NoTsr*sceno, 1)
clIndex <- matrix(rep(NA, NoTsr*sceno), NoTsr*sceno, 1)
```

```
clMASS0.05 <- matrix(rep(NA, NoTsr*sceno), NoTsr*sceno, 1)
clMASS0.1 <- matrix(rep(NA, NoTsr*sceno), NoTsr*sceno, 1)
clMASS0.15 <- matrix(rep(NA, NoTsr*sceno), NoTsr*sceno, 1)
clMASS0.2 <- matrix(rep(NA, NoTsr*sceno), NoTsr*sceno, 1)
clMASS0.25 <- matrix(rep(NA, NoTsr*sceno), NoTsr*sceno, 1)
clMASS0.3 <- matrix(rep(NA, NoTsr*sceno), NoTsr*sceno, 1)
clMASS0.35 <- matrix(rep(NA, NoTsr*sceno), NoTsr*sceno, 1)
clMASS0.4 <- matrix(rep(NA, NoTsr*sceno), NoTsr*sceno, 1)
clMASS0.45 <- matrix(rep(NA, NoTsr*sceno), NoTsr*sceno, 1)
clMASS0.5 <- matrix(rep(NA, NoTsr*sceno), NoTsr*sceno, 1)
clMASS0.55 <- matrix(rep(NA, NoTsr*sceno), NoTsr*sceno, 1)
clMASS0.6 <- matrix(rep(NA, NoTsr*sceno), NoTsr*sceno, 1)
clMASS0.65 <- matrix(rep(NA, NoTsr*sceno), NoTsr*sceno, 1)
clMASS0.7 <- matrix(rep(NA, NoTsr*sceno), NoTsr*sceno, 1)
clMASS0.75 <- matrix(rep(NA, NoTsr*sceno), NoTsr*sceno, 1)
clMASS0.8 <- matrix(rep(NA, NoTsr*sceno), NoTsr*sceno, 1)
clMASS0.85 <- matrix(rep(NA, NoTsr*sceno), NoTsr*sceno, 1)
clMASS0.9 <- matrix(rep(NA, NoTsr*sceno), NoTsr*sceno, 1)
clMASS0.95 <- matrix(rep(NA, NoTsr*sceno), NoTsr*sceno, 1)
clMASS1.0 <- matrix(rep(NA, NoTsr*sceno), NoTsr*sceno, 1)


for (H in H_seq){
  for (RGG in R_seq){
    scecount = scecount + 1
    print(scecount)
    print(RGG)

    # a matrix for dgTSS 9 rows (Tsr 0.1-0.9), 1000 columns (1000 rep for each Tsr)
    TSSmat <- matrix(rep(NA, NoTsr * repno), NoTsr, repno)
    DNAmat <- matrix(rep(NA, NoTsr * repno), NoTsr, repno)
    Indexmat <- matrix(rep(NA, NoTsr * repno), NoTsr, repno)
    MASS0.05mat <- matrix(rep(NA, NoTsr * repno), NoTsr, repno)
    MASS0.1mat <- matrix(rep(NA, NoTsr * repno), NoTsr, repno)
    MASS0.15mat <- matrix(rep(NA, NoTsr * repno), NoTsr, repno)
    MASS0.2mat <- matrix(rep(NA, NoTsr * repno), NoTsr, repno)
    MASS0.25mat <- matrix(rep(NA, NoTsr * repno), NoTsr, repno)
    MASS0.3mat <- matrix(rep(NA, NoTsr * repno), NoTsr, repno)
    MASS0.35mat <- matrix(rep(NA, NoTsr * repno), NoTsr, repno)
    MASS0.4mat <- matrix(rep(NA, NoTsr * repno), NoTsr, repno)
    MASS0.45mat <- matrix(rep(NA, NoTsr * repno), NoTsr, repno)
    MASS0.5mat <- matrix(rep(NA, NoTsr * repno), NoTsr, repno)
    MASS0.55mat <- matrix(rep(NA, NoTsr * repno), NoTsr, repno)
    MASS0.6mat <- matrix(rep(NA, NoTsr * repno), NoTsr, repno)
```

```
MASS0.65mat <- matrix(rep(NA, NoTsr * repno), NoTsr, repno)
MASS0.7mat <- matrix(rep(NA, NoTsr * repno), NoTsr, repno)
MASS0.75mat <- matrix(rep(NA, NoTsr * repno), NoTsr, repno)
MASS0.8mat <- matrix(rep(NA, NoTsr * repno), NoTsr, repno)
MASS0.85mat <- matrix(rep(NA, NoTsr * repno), NoTsr, repno)
MASS0.9mat <- matrix(rep(NA, NoTsr * repno), NoTsr, repno)
MASS0.95mat <- matrix(rep(NA, NoTsr * repno), NoTsr, repno)
MASS1.0mat <- matrix(rep(NA, NoTsr * repno), NoTsr, repno)

# generate common original data for given H, R (VE, VQ, gq, etc.)
ori_scen <- datageneration(H, RGG, info)

# reiteration
for (i in 1:repno){
  # generate g, p and conduct one selection for given H, R
  ori_data <- sort(info, ori_scen)

  for (Tsr in Tsr_seq){
    sel1 <- selection(info, ori_scen, ori_data,Tsr)

    TSSmat[Tsr*20, i] <- sel1$dgTSS
    DNAmat[Tsr*20, i] <- sel1$dgDNA
    Indexmat[Tsr*20, i] <- sel1$dgIndex
    MASS0.05mat[Tsr*20, i] <- sel1$dgMASS[1]
    MASS0.1mat[Tsr*20, i] <- sel1$dgMASS[2]
    MASS0.15mat[Tsr*20, i] <- sel1$dgMASS[3]
    MASS0.2mat[Tsr*20, i] <- sel1$dgMASS[4]
    MASS0.25mat[Tsr*20, i] <- sel1$dgMASS[5]
    MASS0.3mat[Tsr*20, i] <- sel1$dgMASS[6]
    MASS0.35mat[Tsr*20, i] <- sel1$dgMASS[7]
    MASS0.4mat[Tsr*20, i] <- sel1$dgMASS[8]
    MASS0.45mat[Tsr*20, i] <- sel1$dgMASS[9]
    MASS0.5mat[Tsr*20, i] <- sel1$dgMASS[10]
    MASS0.55mat[Tsr*20, i] <- sel1$dgMASS[11]
    MASS0.6mat[Tsr*20, i] <- sel1$dgMASS[12]
    MASS0.65mat[Tsr*20, i] <- sel1$dgMASS[13]
    MASS0.7mat[Tsr*20, i] <- sel1$dgMASS[14]
    MASS0.75mat[Tsr*20, i] <- sel1$dgMASS[15]
    MASS0.8mat[Tsr*20, i] <- sel1$dgMASS[16]
    MASS0.85mat[Tsr*20, i] <- sel1$dgMASS[17]
    MASS0.9mat[Tsr*20, i] <- sel1$dgMASS[18]
    MASS0.95mat[Tsr*20, i] <- sel1$dgMASS[19]
    MASS1.0mat[Tsr*20, i] <- sel1$dgMASS[20]
  }
```

```
}# end of reiteration for (i in 1:repno){}

# calculate means of each selection proportion
for (j in 1:NoTsr){ # j is the jth Tsr e.g. j = 1, Tsr = 0.1, j = 9, Tsr = 0.9
  Hmat[NoTsr * (scecount - 1) + j, 1] <- H # a matrix of H
  Rmat[NoTsr * (scecount - 1) + j, 1] <- RGG # a matrix of R
  Tsrmat[NoTsr * (scecount - 1) + j, 1] <- Tsr_seq[j] # a matrix of Tsr

  mTSS[NoTsr * (scecount - 1) + j, 1] <- mean(TSSmat[j, ])
  mDNA[NoTsr * (scecount - 1) + j, 1] <- mean(DNAmat[j, ])
  mIndex[NoTsr * (scecount - 1) + j, 1] <- mean(Indexmat[j, ])
  mMASS0.05[NoTsr * (scecount - 1) + j, 1] <- mean(MASS0.05mat[j, ])
  mMASS0.1[NoTsr * (scecount - 1) + j, 1] <- mean(MASS0.1mat[j, ])
  mMASS0.15[NoTsr * (scecount - 1) + j, 1] <- mean(MASS0.15mat[j, ])
  mMASS0.2[NoTsr * (scecount - 1) + j, 1] <- mean(MASS0.2mat[j, ])
  mMASS0.25[NoTsr * (scecount - 1) + j, 1] <- mean(MASS0.25mat[j, ])
  mMASS0.3[NoTsr * (scecount - 1) + j, 1] <- mean(MASS0.3mat[j, ])
  mMASS0.35[NoTsr * (scecount - 1) + j, 1] <- mean(MASS0.35mat[j, ])
  mMASS0.4[NoTsr * (scecount - 1) + j, 1] <- mean(MASS0.4mat[j, ])
  mMASS0.45[NoTsr * (scecount - 1) + j, 1] <- mean(MASS0.45mat[j, ])
  mMASS0.5[NoTsr * (scecount - 1) + j, 1] <- mean(MASS0.5mat[j, ])
  mMASS0.55[NoTsr * (scecount - 1) + j, 1] <- mean(MASS0.55mat[j, ])
  mMASS0.6[NoTsr * (scecount - 1) + j, 1] <- mean(MASS0.6mat[j, ])
  mMASS0.65[NoTsr * (scecount - 1) + j, 1] <- mean(MASS0.65mat[j, ])
  mMASS0.7[NoTsr * (scecount - 1) + j, 1] <- mean(MASS0.7mat[j, ])
  mMASS0.75[NoTsr * (scecount - 1) + j, 1] <- mean(MASS0.75mat[j, ])
  mMASS0.8[NoTsr * (scecount - 1) + j, 1] <- mean(MASS0.8mat[j, ])
  mMASS0.85[NoTsr * (scecount - 1) + j, 1] <- mean(MASS0.85mat[j, ])
  mMASS0.9[NoTsr * (scecount - 1) + j, 1] <- mean(MASS0.9mat[j, ])
  mMASS0.95[NoTsr * (scecount - 1) + j, 1] <- mean(MASS0.95mat[j, ])
  mMASS1.0[NoTsr * (scecount - 1) + j, 1] <- mean(MASS1.0mat[j, ])

  varTSS[NoTsr * (scecount - 1) + j, 1] <- var(TSSmat[j, ])
  varDNA[NoTsr * (scecount - 1) + j, 1] <- var(DNAmat[j, ])
  varIndex[NoTsr * (scecount - 1) + j, 1] <- var(Indexmat[j, ])
  varMASS0.05[NoTsr * (scecount - 1) + j, 1] <- var(MASS0.05mat[j, ])
  varMASS0.1[NoTsr * (scecount - 1) + j, 1] <- var(MASS0.1mat[j, ])
  varMASS0.15[NoTsr * (scecount - 1) + j, 1] <- var(MASS0.15mat[j, ])
  varMASS0.2[NoTsr * (scecount - 1) + j, 1] <- var(MASS0.2mat[j, ])
  varMASS0.25[NoTsr * (scecount - 1) + j, 1] <- var(MASS0.25mat[j, ])
  varMASS0.3[NoTsr * (scecount - 1) + j, 1] <- var(MASS0.3mat[j, ])
  varMASS0.35[NoTsr * (scecount - 1) + j, 1] <- var(MASS0.35mat[j, ])
  varMASS0.4[NoTsr * (scecount - 1) + j, 1] <- var(MASS0.4mat[j, ])
```

```
varMASS0.45[NoTsr * (scecount - 1) + j, 1] <- var(MASS0.45mat[j, ])
varMASS0.5[NoTsr * (scecount - 1) + j, 1] <- var(MASS0.5mat[j, ])
varMASS0.55[NoTsr * (scecount - 1) + j, 1] <- var(MASS0.55mat[j, ])
varMASS0.6[NoTsr * (scecount - 1) + j, 1] <- var(MASS0.6mat[j, ])
varMASS0.65[NoTsr * (scecount - 1) + j, 1] <- var(MASS0.65mat[j, ])
varMASS0.7[NoTsr * (scecount - 1) + j, 1] <- var(MASS0.7mat[j, ])
varMASS0.75[NoTsr * (scecount - 1) + j, 1] <- var(MASS0.75mat[j, ])
varMASS0.8[NoTsr * (scecount - 1) + j, 1] <- var(MASS0.8mat[j, ])
varMASS0.85[NoTsr * (scecount - 1) + j, 1] <- var(MASS0.85mat[j, ])
varMASS0.9[NoTsr * (scecount - 1) + j, 1] <- var(MASS0.9mat[j, ])
varMASS0.95[NoTsr * (scecount - 1) + j, 1] <- var(MASS0.95mat[j, ])
varMASS1.0[NoTsr * (scecount - 1) + j, 1] <- var(MASS1.0mat[j, ])

clTSS[NoTsr * (scecount - 1) + j, 1] <- 1.96 * (varTSS[NoTsr * (scecount - 1) + j,1]^ 0.5 /1000 ^ 0.5)
clDNA[NoTsr * (scecount - 1) + j, 1] <- 1.96 * (varDNA[NoTsr * (scecount - 1) + j,1]^ 0.5 /1000 ^
0.5)
clIndex[NoTsr * (scecount - 1) + j, 1] <- 1.96 * (varIndex[NoTsr * (scecount - 1) + j,1]^ 0.5 /1000 ^
0.5)
clMASS0.05[NoTsr * (scecount - 1) + j, 1] <- 1.96 * (varMASS0.05[NoTsr * (scecount - 1) + j,1]^
0.5 /1000 ^ 0.5)
clMASS0.1[NoTsr * (scecount - 1) + j, 1] <- 1.96 * (varMASS0.1[NoTsr * (scecount - 1) + j,1]^ 0.5
/1000 ^ 0.5)
clMASS0.15[NoTsr * (scecount - 1) + j, 1] <- 1.96 * (varMASS0.15[NoTsr * (scecount - 1) + j,1]^
0.5 /1000 ^ 0.5)
clMASS0.2[NoTsr * (scecount - 1) + j, 1] <- 1.96 * (varMASS0.2[NoTsr * (scecount - 1) + j,1]^ 0.5
/1000 ^ 0.5)
clMASS0.25[NoTsr * (scecount - 1) + j, 1] <- 1.96 * (varMASS0.25[NoTsr * (scecount - 1) + j,1]^
0.5 /1000 ^ 0.5)
clMASS0.3[NoTsr * (scecount - 1) + j, 1] <- 1.96 * (varMASS0.3[NoTsr * (scecount - 1) + j,1]^ 0.5
/1000 ^ 0.5)
clMASS0.35[NoTsr * (scecount - 1) + j, 1] <- 1.96 * (varMASS0.35[NoTsr * (scecount - 1) + j,1]^
0.5 /1000 ^ 0.5)
clMASS0.4[NoTsr * (scecount - 1) + j, 1] <- 1.96 * (varMASS0.4[NoTsr * (scecount - 1) + j,1]^ 0.5
/1000 ^ 0.5)
clMASS0.45[NoTsr * (scecount - 1) + j, 1] <- 1.96 * (varMASS0.45[NoTsr * (scecount - 1) + j,1]^
0.5 /1000 ^ 0.5)
clMASS0.5[NoTsr * (scecount - 1) + j, 1] <- 1.96 * (varMASS0.5[NoTsr * (scecount - 1) + j,1]^ 0.5
/1000 ^ 0.5)
clMASS0.55[NoTsr * (scecount - 1) + j, 1] <- 1.96 * (varMASS0.55[NoTsr * (scecount - 1) + j,1]^
0.5 /1000 ^ 0.5)
clMASS0.6[NoTsr * (scecount - 1) + j, 1] <- 1.96 * (varMASS0.6[NoTsr * (scecount - 1) + j,1]^ 0.5
/1000 ^ 0.5)
clMASS0.65[NoTsr * (scecount - 1) + j, 1] <- 1.96 * (varMASS0.65[NoTsr * (scecount - 1) + j,1]^
0.5 /1000 ^ 0.5)
```

```
      clMASS0.7[NoTsr * (scecount - 1) + j, 1] <- 1.96 * (varMASS0.7[NoTsr * (scecount - 1) + j,1]^ 0.5
/1000 ^ 0.5)
      clMASS0.75[NoTsr * (scecount - 1) + j, 1] <- 1.96 * (varMASS0.75[NoTsr * (scecount - 1) + j,1]^
0.5 /1000 ^ 0.5)
      clMASS0.8[NoTsr * (scecount - 1) + j, 1] <- 1.96 * (varMASS0.8[NoTsr * (scecount - 1) + j,1]^ 0.5
/1000 ^ 0.5)
      clMASS0.85[NoTsr * (scecount - 1) + j, 1] <- 1.96 * (varMASS0.85[NoTsr * (scecount - 1) + j,1]^
0.5 /1000 ^ 0.5)
      clMASS0.9[NoTsr * (scecount - 1) + j, 1] <- 1.96 * (varMASS0.9[NoTsr * (scecount - 1) + j,1]^ 0.5
/1000 ^ 0.5)
      clMASS0.95[NoTsr * (scecount - 1) + j, 1] <- 1.96 * (varMASS0.95[NoTsr * (scecount - 1) + j,1]^
0.5 /1000 ^ 0.5)
      clMASS1.0[NoTsr * (scecount - 1) + j, 1] <- 1.96 * (varMASS1.0[NoTsr * (scecount - 1) + j,1]^ 0.5
/1000 ^ 0.5)
    }
  }
}



result <- list('H' = Hmat, 'R' = Rmat, 'Tsr' = Tsrmat,
              'TSS' = mTSS, 'TSSvar' = varTSS, 'TSScl' = clTSS,
              'DNA' = mDNA, 'DNAvar' = varDNA, 'DNAcl' = clDNA,
              'Index' = mIndex, 'Indexvar' = varIndex, 'Indexcl' = clIndex,
              'MASS0.05' = mMASS0.05,'var0.05' = varMASS0.05,'cl0.05' = clMASS0.05,
              'MASS0.1' = mMASS0.1,'var0.1' = varMASS0.1,'cl0.1' = clMASS0.1,
              'MASS0.15' = mMASS0.15,'var0.15' = varMASS0.15,'cl0.15' = clMASS0.15,
              'MASS0.2' = mMASS0.2,'var0.2' = varMASS0.2,'cl0.2' = clMASS0.2,
              'MASS0.25' = mMASS0.25,'var0.25' = varMASS0.25,'cl0.25' = clMASS0.25,
              'MASS0.3' = mMASS0.3,'var0.3' = varMASS0.3,'cl0.3' = clMASS0.3,
              'MASS0.35' = mMASS0.35,'var0.35' = varMASS0.35,'cl0.35' = clMASS0.35,
              'MASS0.4' = mMASS0.4,'var0.4' = varMASS0.4,'cl0.4' = clMASS0.4,
              'MASS0.45' = mMASS0.45,'var0.45' = varMASS0.45,'cl0.45' = clMASS0.45,
              'MASS0.5' = mMASS0.5,'var0.5' = varMASS0.5,'cl0.5' = clMASS0.5,
              'MASS0.55' = mMASS0.55,'var0.55' = varMASS0.55,'cl0.55' = clMASS0.55,
              'MASS0.6' = mMASS0.6,'var0.6' = varMASS0.6,'cl0.6' = clMASS0.6,
              'MASS0.65' = mMASS0.65,'var0.65' = varMASS0.65,'cl0.65' = clMASS0.65,
              'MASS0.7' = mMASS0.7,'var0.7' = varMASS0.7,'cl0.7' = clMASS0.7,
              'MASS0.75' = mMASS0.75,'var0.75' = varMASS0.75,'cl0.75' = clMASS0.75,
              'MASS0.8' = mMASS0.8,'var0.8' = varMASS0.8,'cl0.8' = clMASS0.8,
              'MASS0.85' = mMASS0.85,'var0.85' = varMASS0.85,'cl0.85' = clMASS0.85,
              'MASS0.9' = mMASS0.9,'var0.9' = varMASS0.9,'cl0.9' = clMASS0.9,
              'MASS0.95' = mMASS0.95,'var0.95' = varMASS0.95,'cl0.95' = clMASS0.95,
              'MASS1.0' = mMASS1.0,'var1.0' = varMASS1.0,'cl1.0' = clMASS1.0)
```

```
write.csv(result, file = sprintf("AllHR_030315.csv"), row.names = F)
```
